# *Cannabis*: a multifaceted plant with endless potentials

**DOI:** 10.3389/fphar.2023.1200269

**Published:** 2023-06-15

**Authors:** Eric Fordjour, Charles F. Manful, Albert A. Sey, Rabia Javed, Thu Huong Pham, Raymond Thomas, Mumtaz Cheema

**Affiliations:** ^1^ School of Science and the Environment, Memorial University of Newfoundland, Corner Brook, NL, Canada; ^2^ Biotron Experimental Climate Change Research Centre/Department of Biology, University of Western Ontario, London, ON, Canada

**Keywords:** marijuana, cannabiniods, pharmacology, ethnobotany, phytochemistry

## Abstract

*Cannabis sativa*, also known as “hemp” or “weed,” is a versatile plant with various uses in medicine, agriculture, food, and cosmetics. This review attempts to evaluate the available literature on the ecology, chemical composition, phytochemistry, pharmacology, traditional uses, industrial uses, and toxicology of *Cannabis sativa*. So far, 566 chemical compounds have been isolated from *Cannabis*, including 125 cannabinoids and 198 non-cannabinoids. The psychoactive and physiologically active part of the plant is a cannabinoid, mostly found in the flowers, but also present in smaller amounts in the leaves, stems, and seeds. Of all phytochemicals, terpenes form the largest composition in the plant. Pharmacological evidence reveals that the plants contain cannabinoids which exhibit potential as antioxidants, antibacterial agents, anticancer agents, and anti-inflammatory agents. Furthermore, the compounds in the plants have reported applications in the food and cosmetic industries. Significantly, *Cannabis* cultivation has a minimal negative impact on the environment in terms of cultivation. Most of the studies focused on the chemical make-up, phytochemistry, and pharmacological effects, but not much is known about the toxic effects. Overall, the *Cannabis* plant has enormous potential for biological and industrial uses, as well as traditional and other medicinal uses. However, further research is necessary to fully understand and explore the uses and beneficial properties of *Cannabis sativa*.

## 1 Introduction

Throughout human civilization, there has been a pursuit of plants for their unique potential, including medicinal use. Evidence of this dates back to 60,000 years, with a recent discovery of a 5,000-year-old Sumerian clay tablet that confirms the use of medicinal plants in drug production (Sumner, 2000). Natural resources like medicinal plants, also known as green medicine, are gaining popularity worldwide due to their safety, effectiveness, cultural acceptance, and lower risk of adverse effects compared to synthetic medications (Mustafa et al., 2017). Today, traditional botanical medicines are widely used to treat human health problems, with over 80% of the global population depending on them (Mander and Liu, 2010).

*Cannabis sativa* L. (2n ¼ 20) is a well-known plant that has been around since the beginning of time (Small, 2017). This annual plant is a member of the family Cannabaceae and a widespread plant found in varied environments (Andre et al., 2016). It has been used by humans for over 5,000 years and is one of the oldest plant sources of food and fiber (Appendino et al., 2008). The botanical types of *Cannabis sativa* differ in terms of their chemical content, plant growth habits, agronomic requirements, and processing (Datwyler and Weiblen, 2006). *Cannabis* flowers and leaves have a distinctive aroma, and the plant’s extracts include a variety of beneficial flavonoids, terpenes, and other compounds that are efficient insecticides, fungicides, and therapeutic agents (Pellati et al., 2018). The flower, leaves, oil, and trichome of the plant have been shown to be cytotoxic, antimicrobial, antioxidant, antihypertensive, antipyretic, and appetite-stimulating (Russo and Marcu, 2017). The flower extracts with antioxidant activity have been shown to have health-promoting and anti-aging properties, and are utilized to treat a variety of metabolic and chronic disorders, including glaucoma, pain, depression, cancer, liver disease, cardiovascular diseases, inflammation, and metabolic syndrome (Nallathambi et al., 2017). As an agricultural crop, industrial *Cannabis* (hemp), is a plant that may be harvested for its fiber (Johnson, 2014). While in the cosmetic industry, it is used for skincare products such as anti-aging creams and hair food (Schettino et al., 2021). Traditionally, the seeds are used for making oil, while the leaves were the second most consumed part of the plant and were used in various ways, such as seasoning, baking, flour, and added to meals (Iftikhar et al., 2021; Kuppuram, 2022; Xu et al., 2022).

Even though *Cannabis* is used in many ways, the drug’s unclear legal status worldwide has made it hard to study for the last century (Smith et al., 2014). In addition, there has not been much information about comprehensive analysis of the plant that can show the plant’s usefulness in all aspects. In this review, *Cannabis*’ potential is discussed in length to provide thorough and up-to-date information on the *Cannabis* plants.

## 2 Ethnobotany of *Cannabis*

### 2.1 Ecology and distribution

*Cannabis sativa’s* origin is unknown, but it is believed to have come from temperate regions in Asia, specifically the southern Caspian region, Siberia, China, or the Himalayas (Minelli, 2015). However, due to widespread transportation and modification by humans over the past 6,000 years, it is challenging to determine its original geographic range or whether a plant collected in nature is a primitive wild type or has been influenced by human domestication (Sharma, 1979). “Weed” is the most common informal name for the marijuana form of *Cannabis sativa*, and it accurately describes the species as a weed that grows primarily in habitats created or modified by humans (Small, 2015). It can be found in various places such as fields, trash heaps, vacant lots, pastures, ditches, creeks, and open woods. However, it is poorly adapted to infiltrating established perennial stands and typically invades only after the soil has been recently disturbed or plowed (Small, 2017).

Except in drainage channels, where it is extremely well suited, weedy *Cannabis sativa* is a slow colonizer, spreading slowly throughout the landscape. It is possible to judge the ecology of *Cannabis sativa* prior to human intervention based on the circumstances and adaptations of existing wild-growing populations of this plant species (Small, 2017). By examining the circumstances and adaptations of these populations, researchers can gain insight into the plant’s natural habitat, growth patterns, and environmental interactions. For example, studying the genetic diversity of wild-growing *Cannabis sativa* populations can provide information on the plant’s evolutionary history and geographic distribution (Small, 2015). Additionally, analyzing the physical characteristics of wild *Cannabis sativa* plants, such as their size, leaf shape, and stem structure, can provide clues about their adaptation to various environmental conditions (Ren et al., 2021).

### 2.2 Taxonomic classification and common names

**FIGURE 1:**
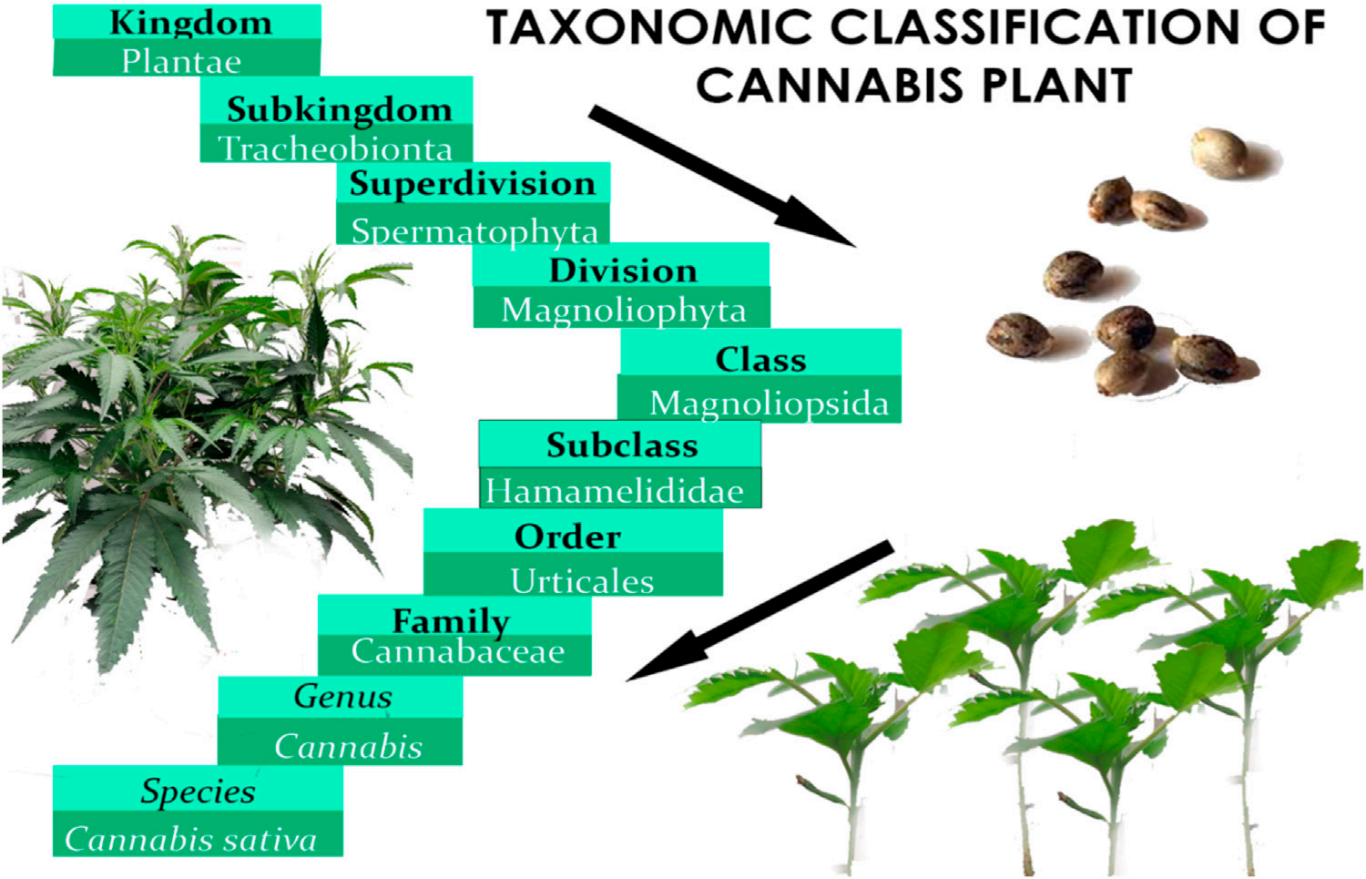
Taxonomic classification of *Cannabis* plant.

**FIGURE 2:**
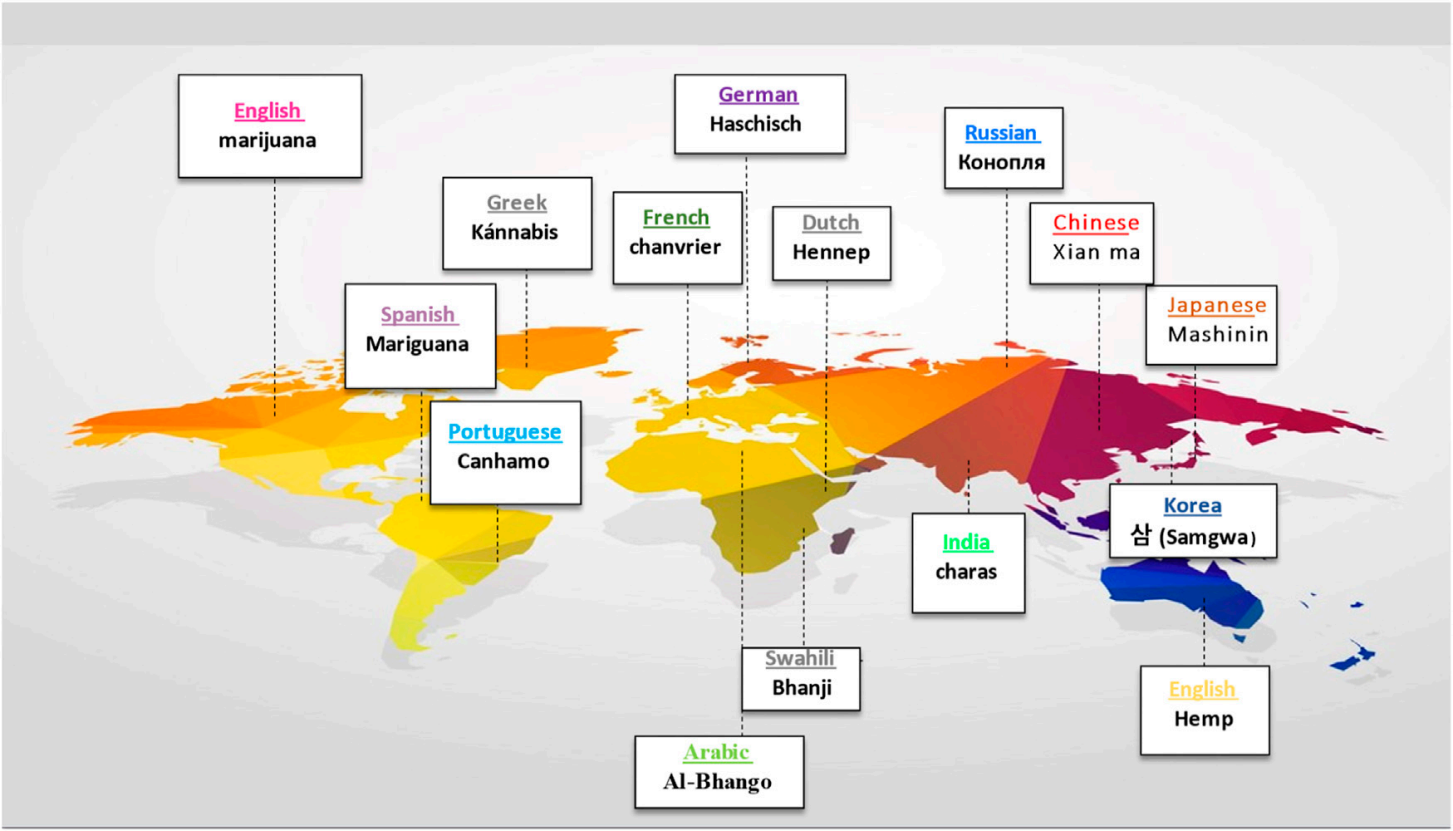
The name of *Cannabis* in some popular languages.

Before Linnaeus published Species Plantarum in the 18th century, domestic hemp was known by various names, including *Cannabis angustifolia*, *Cannabis sativa*, and *Cannabis indica* (Linnaeus, 1753). Later, Jean-Baptiste Lamarck proposed a division between extensively cultivated *Cannabis* species in western continents and the wild variety found in India (Erkelens and Hazekamp, 2014). After 50 years, Lindley reclassified *Cannabis* under Linnaeus’ classification system, affirming the plant’s monospecific status for the rest of the century (Lindley, 2011). Below is the botanical classification of *Cannabis* plant (Figure 1) (Small, 2017). In the early 20th century, a new species called *Cannabis ruderalis* emerged, but it was not until 1975 that the restoration of the *Cannabis indica* species to its current name was proposed (Holland, 2010). Figure 2 presents the name of *Cannabis* in some popular languages.

*Cannabis* is a polymorphic plant, and chemotaxonomic markers are effective in differentiating between different *Cannabis* germplasms and screening for hybrids (Piomelli and Russo, 2016). Small and Cronquist (1976) used biphasic techniques (use of distinct approaches) to identify the four subspecies of *Cannabis sativa*, including *sativa* var. *sativa*, *sativa* var. *spontanea*, *indica* var. *indica*, and *indica* var*. kafiristanica* based on morphological and chemical characteristics such as fruit morphology and THC content (Schultes et al., 1975; Pollio, 2016). Both variants of the subspecies *sativa* are widely cultivated in North America, Europe, and Asia, and have low intoxicating potential when compared to other *Cannabis* cultivars (Small and Cronquist, 1976). Meanwhile, the subspecies Indica’s variants have a strong intoxicating potential and are primarily found in the Asiatic Continent (Pollio, 2016; Small, 2017).

### 2.3 Legality-based classification

Despite being an arbitrary term that does not reflect the drug’s properties, *Cannabis* is classified as a “narcotic” (i.e., illegal drug) in the legal world (Small, 2017). An illegal drug is defined as a chemical or preparation associated with severe punishments due to its actual or suspected detrimental properties (Smith et al., 2014). *Cannabis* has been criminalized since the Second World War due to its popular use as a recreational substance, leading to limited research and commercial development in the sector. As a result, research and commercial development on the plant was prohibited for most of the 20th century (Appendino et al., 2014). *Cannabis sativa* became the most commonly cultivated black market crop in the Western world after World War II, leading to the allocation of significant law enforcement resources to remove the plants (Chouvy, 2019). Scientific investigations in Western countries were mostly approved for criminal justice-related forensic studies to assist law enforcement or medical and social-related studies to document and alleviate negative consequences (Chandra et al., 2008).

Criminalizing *Cannabis* has led to high law enforcement costs and social instability, and many jurisdictions are looking to reduce penalties for its possession and consumption (Small, 2015). The legalization of medical *Cannabis* is widely accepted, but recreational use is still under debate (Cruz et al., 2016). While punishments for illegal drug use have softened in several countries due to increased public acceptance, although, capital punishment is still a possibility in some Asian countries (Chandra et al., 2008; Small, 2017). The decriminalization of *Cannabis* use is not unique to the North American continent. More than forty countries have legalized the use of marijuana for medical or recreational purposes. Among these countries are Argentina, Germany, Chile, Colombia, and South Africa (Chouvy, 2019). Additionally, Canada, 18 United States states, and two territories—the District of Columbia and the Australian Capital Territory—have legalized *Cannabis*. New strains are approved for use in Canada until 2023, and Health Canada has issued regulations amending the *Cannabis* Act and *Cannabis* Regulations to ensure proper regulation of *Cannabis* (Canada, 2018; Caplan, 2018).

### 2.4 Therapeutic based classification

The cannabinoids in *Cannabis* are unique terpene phenolic substances. Approximately 100 cannabinoids are produced in epidermal trichomes but in small quantities (Mölleken and Theimer, 1997). As discussed by Small (2017), *Cannabis*’ psychological effects have been ambiguously called “narcotic” in popular, legal, and scientific contexts. *Cannabis* and opioids are legally grouped, but they are pharmacologically distinct. “Narcotic” comes from “narcosis,” a substance that induces sleep, but it is used to refer to any medicine that induces sleep, stupor, or insensibility (Macdonald and Rotermann, 2017). In moderate amounts, psychoactive cannabinoids such as THC and CBD in *Cannabis* can induce sedation (Piomelli and Russo, 2016). CBD has a stimulant effect in low and moderate concentrations, and only in high concentrations has a soothing effect (Piomelli and Russo, 2016). *Cannabis sativa’s* abundant myrcene is likewise sedative (Russo, 2014). There is still some disagreement on how *Cannabis* should be pharmacologically classified (Kalant, 2010). In some cases, *Cannabis* has been classified as a sedative-hypnotic-general anesthetic, a mixed stimulant-depressant, a mild hallucinogen, and a psychedelic (Degenhardt et al., 2015). In surgical and dental procedures, it is referred to as a sedative-hypnotic general anesthetic. *Cannabis*’s psychedelic, hallucinogenic, psychotomimetic, and psychotic properties are misrepresented by terms like “psychedelic” (Brewster, 2019). While “hallucinogenic” is no longer acceptable, “psychoactive,” “euphoric,” or “intoxicating” are the best pharmacological names for *Cannabis* (Small, 2017; Brewster, 2019). According to Troutt and DiDonato (2015), medical *Cannabis* users in the United States are characterized by daily dosing and weekly consumption of 6–9 g (Ko et al., 2016). In Canada, 42% of medical marijuana patients consume 2 to 3 times a day, and 40% consume more than 14 g per week. In Canada and the United States, most patients inhale (Ilgen et al., 2013; Bonn-Miller et al., 2014). Surprisingly, only 53% of adult *Cannabis* users in the United States use *Cannabis* purely for recreational purposes, while 47% use it “in part or totally for medicinal purposes,” and 10% use it solely for medicinal purposes (Ko et al., 2016). Research shows that in 2004, about 4% of Canadians over the age of 14 reported using *Cannabis* in the past year for self-identified medical problems (Schauer et al., 2016). *Cannabis* remains the most commonly used drug globally, with more than 4% of the global population aged 15–64 (approximately 209 million people) using *Cannabis* in 2020, a 23% increase from 170 million in 2010 (Richards et al., 2020). Approximately 27% of Israeli adults consumed *Cannabis* in 2020, making it the country with the highest incidence of *Cannabis* use as of that year. (Bar-Or et al., 2021). Comparatively, the United States has a lower incidence of *Cannabis* use, with approximately 17% of the adult population reported to have consumed *Cannabis* within the same period (Sarvet et al., 2018). In Europe, Czechia has the highest incidence of *Cannabis* use of 11.1% among their adult population (Arnarsson et al., 2018). Forecasts put the global *Cannabis* market at $82.3 billion in 2027, a significant projection of 24.3% with $27.7 billion recorded in 2022 (Chen et al., 2021). The United Nations Office on Drugs and Crime (UNODC) identifies Morocco as the largest producer of ‘psychoactive marijuana plants’ worldwide (Kitchen et al., 2022). However, in terms of revenue generation, the United States leads in terms of the sale of medical *Cannabis*, with an annual total of 10 billion dollars, a significant portion of which comes from therapeutic marijuana (Kilmer and MacCoun, 2017). In Europe, Germany leads in the sale of medical marijuana, with an estimate 87.2 million dollars (Häuser et al., 2018). Between 1995 and 2005, 19 African countries reported the cultivation of *Cannabis* within their borders. In 2005, worldwide, *Cannabis* production was estimated at 42,000 metric tons, with Africa alone accounting for 25% of the total (Akyeampong, 2005).

### 2.5 Morphological characteristics of *Cannabis*

**FIGURE 3:**
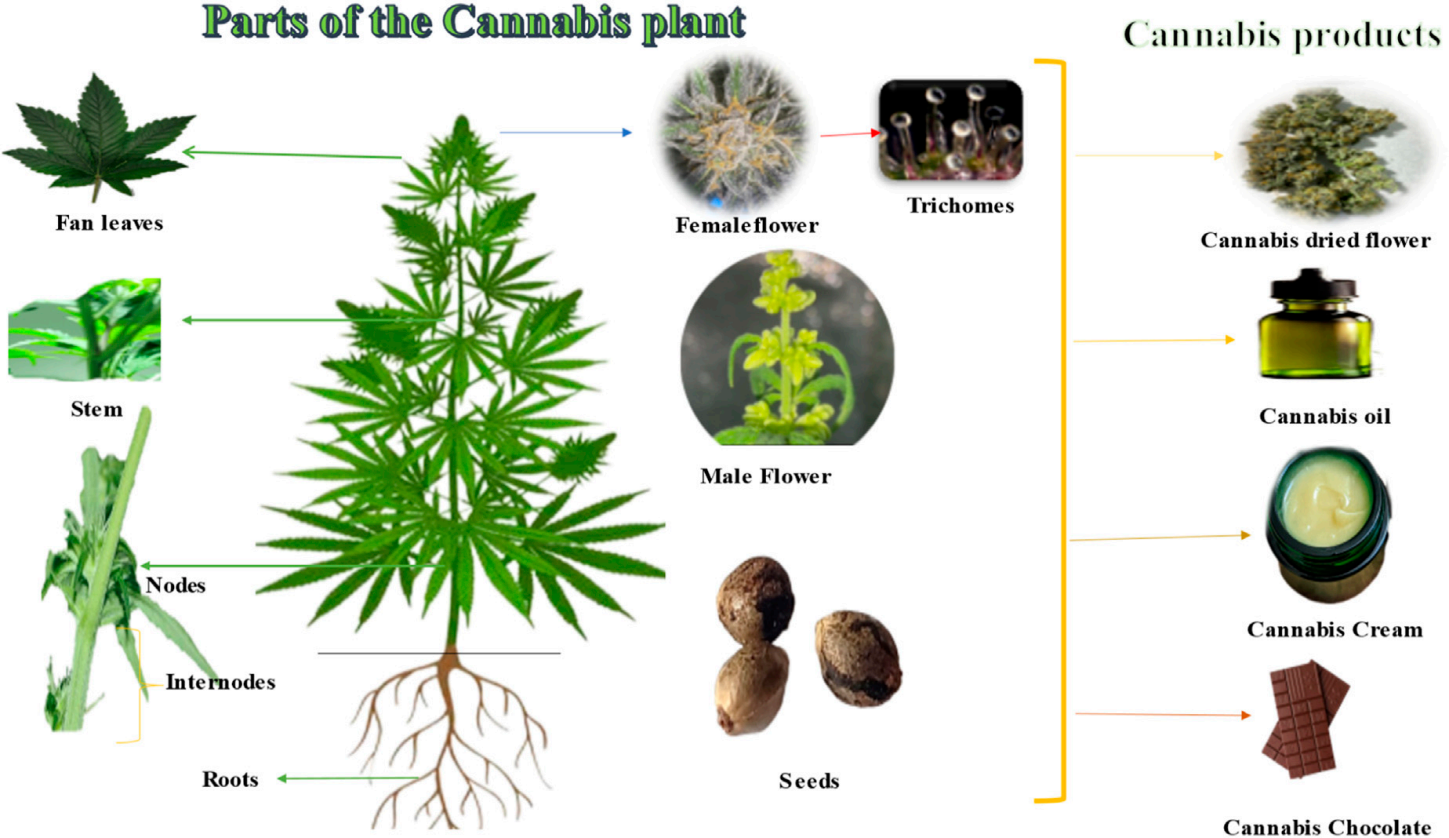
Parts of *Cannabis* plants and products.

*Cannabis sativa* L. is an annual plant that can reach up to 5 m in height and has upright stems with palmate leaves consisting of 5–7 linear-lanceolate leaflets (Figure 3). Male flowers lack petals and grow in axillary or terminal panicles, while female flowers have a single ovule and a perianth that is tightly attached (Farag and Kayser, 2017; Bonini et al., 2018). Trichomes, which are glandular protuberances that cover the plant’s leaves, bracts, and stems, are present in high concentrations (Bonini et al., 2018). The fruit of each flower is a single small smooth light brownish-grey fruit that is then passed on to the next-generation. Female flowers grow at the end of the stem and in the axils. They have one ovule and a perianth that is tightly connected. Male flowers, on the other hand, have five yellowish petals and five anthers (Farag and Kayser, 2017).

## 3 Phytochemistry of *Cannabis*

The number of natural chemicals isolated from *Cannabis sativa* L. has not significantly increased in recent years, despite over 500 compounds being discovered so far (Pellati et al., 2018; Al Ubeed et al., 2022). In 1980, 423 compounds were discovered, which grew to 483 by 1995 (Matsuda et al., 1990; Dos Santos and Romão, 2023). Currently, 566 compounds have been identified and isolated which constitutes over 18 classes of different secondary metabolites found in the plant. These substances have been found to be highly abundant in the flowers and leaves of the plant (Kopustinskiene et al., 2022; Odieka et al., 2022). Out of this number, 125 are cannabinoids, 198 are non-cannabinoids and 120 are terpenes, constituting a total of 443. The rest of the substances identified in the plant in 2021 include 2 alkaloids, 34 flavonoids, 42 phenols and 3 sterols (Al Ubeed et al., 2022). The aromatic quality of female *Cannabis* plants is due to the terpenes they produce, such as pinene, limonene, terpineol, and borneol (McPartland et al., 2001). These terpenes have insect-repellent properties and inhibit the growth of neighboring vegetation. The glandular trichomes on the plant produce a resin that acts as a sophisticated defense mechanism against insects and has the potential to serve as an antibiotic and antifungal agent. These trichomes contain secondary metabolites like phytocannabinoids and terpenoids that are responsible for the plant’s defense and interaction with herbivores and pests, as well as its characteristic scent (Andre et al., 2016). The various phytochemicals are summarized below.

### 3.1 Cannabinoids

Therapeutic marijuana has a high level of tetrahydrocannabinol (THC), but minimal levels of cannabidiolic acid (CBDA) and cannabidiol (CBD). Cannabinoids undergo decarboxylation during drying, storage, and thermal processing, converting from an acidic to a neutral state. There are now many types of cannabinoids, not just those found in *Cannabis*, and the term “phytocannabinoids” has been used for those that naturally come from the plant (Radwan et al., 2021). A total of 120 phytocannabinoids have been identified and divided into 11 categories (Berman et al., 2018; Bonn-Miller et al., 2018). Table 1 lists the 11 subclasses of 120 phytocannabinoids.

**TABLE 1:** Phytocannabinoids discovered in *Cannabis sativa* L.

No.	Cannabinoids	Molecular structure	Reference
1	(–) - Δ^9^-trans-tetrahydrocannabinol (Δ^9^-THC)	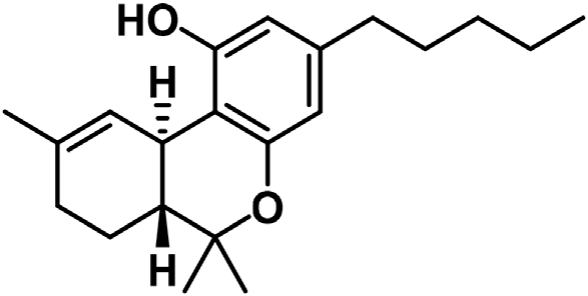	Gaoni and Mechoulam (1964)
2	(–)-Δ^8^-*trans*-tetrahydrocannabinol (Δ^8^-THC)	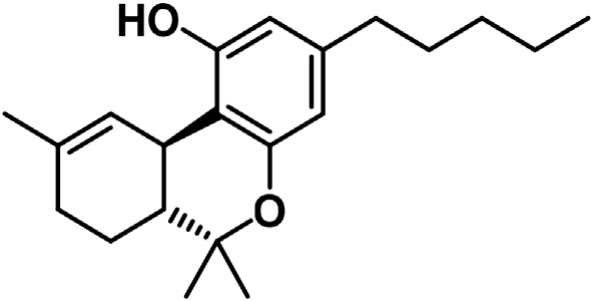	Karniol and Carlini (1973)
3	Cannabigerol (CBG)	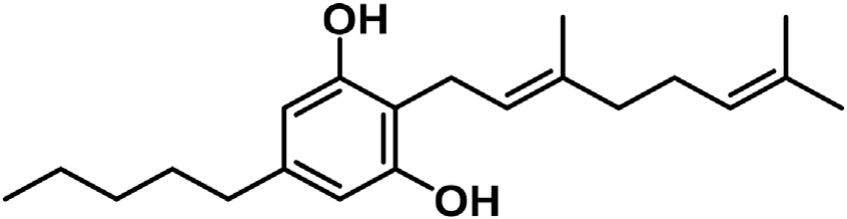	Gülck and Møller (2020)
4	Cannabichromene (CBC)	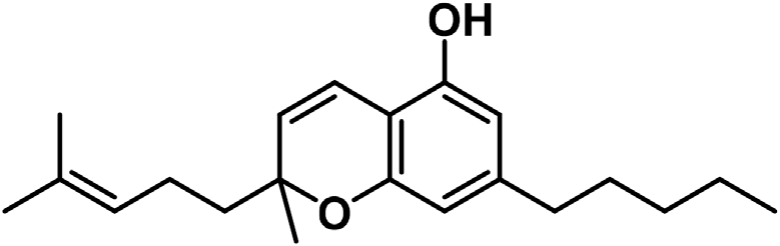	DeLong et al. (2010)
5	Cannabidiol (CBD)	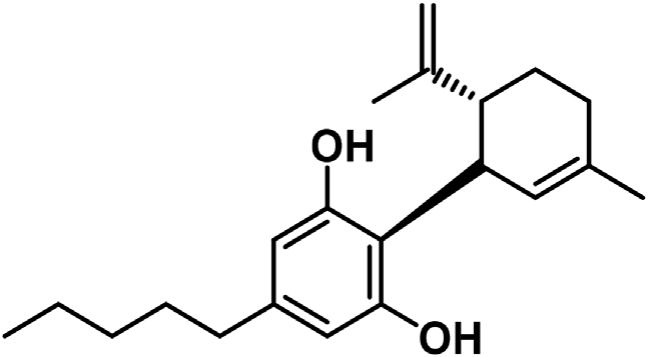	Seltzer et al. (2020)
6	Cannabinodiol (CBND)	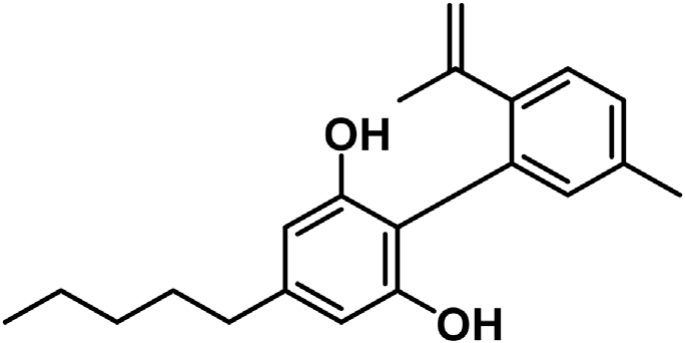	Crippa et al. (2018)
7	Cannabielsoin (CBE)	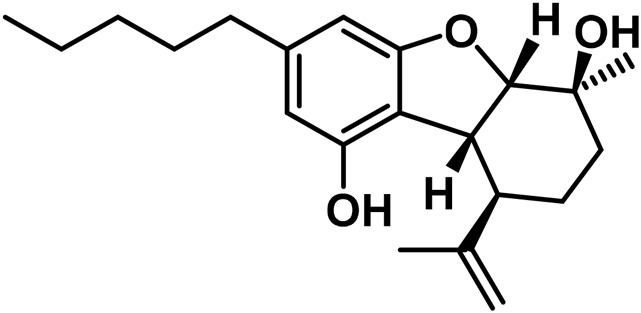	Braemer and Paris (1987)
8	Cannabicyclol (CBL)	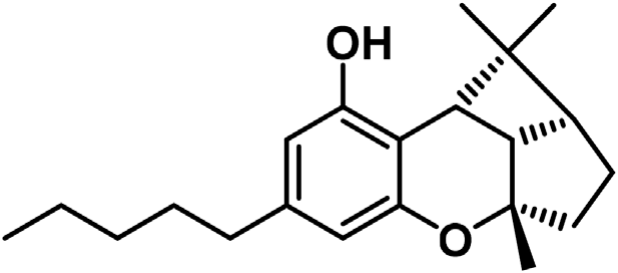	ElSohly and Gul (2014)
9	Cannabinol (CBN)	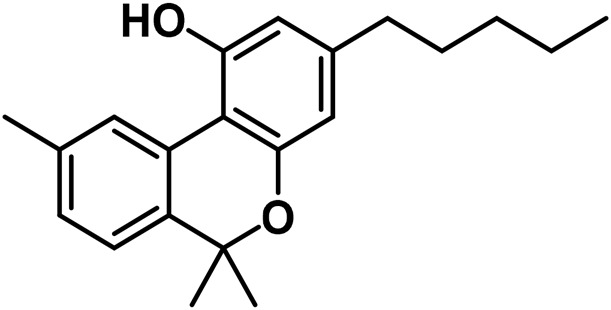	Ross and ElSohly (1997)
10	Cannabitriol (CBT)	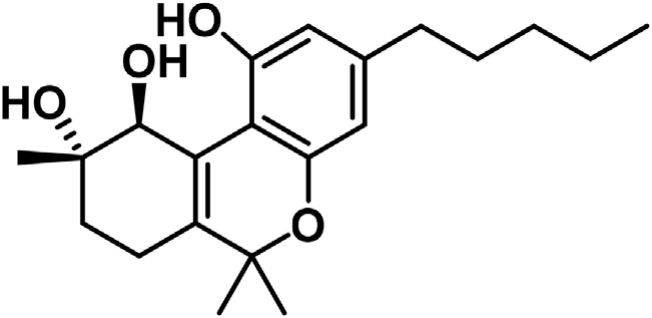	ElSohly and Slade (2005)
11	Miscellaneous types		
i	Dehydrocannabifuran (DCBF-C_5_)	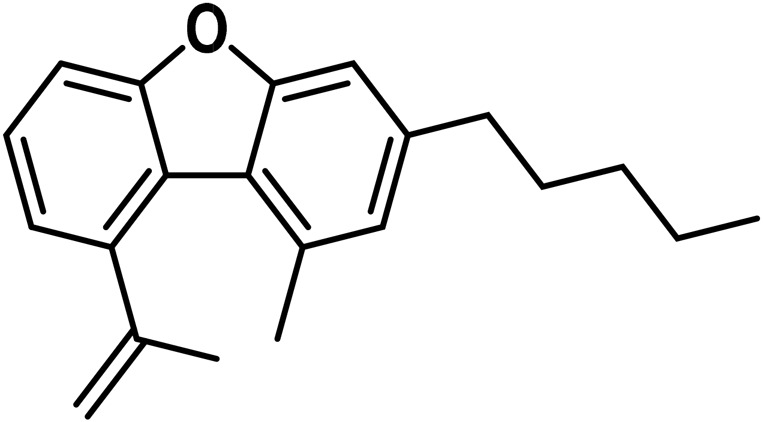	Hanus et al. (2016)
ii	Cannabifuran (CBF-C_5_)	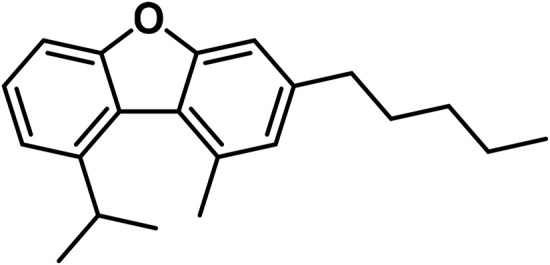	Piccolella et al. (2020)
iii	Cannabichromanone (CBCN-C_5_)	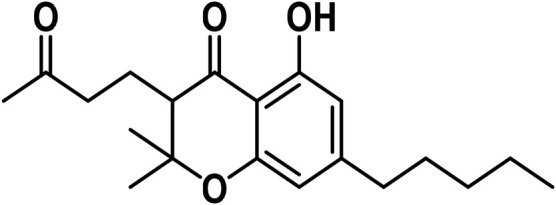	ElSohly and Slade (2005)
iv	Bisnor-Cannabichromanone (CBCN-C_3_)	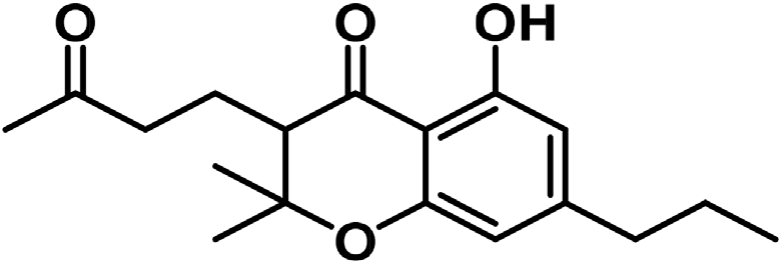	ElSohly and Gul (2014)
v	Cannabicoumaronone (CBCON-C_5_)	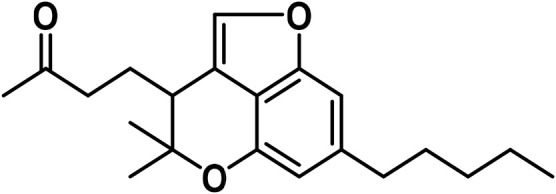	ElSohly and Gul (2014)

#### 3.1.1 (−)-Delta-9-trans-tetrahydrocannabinol (Δ^9^-THC) type

Gaoni and Mechoulam (1971) discovered the structure of Δ^9^-THC and explained its psychoactive properties. Rhee et al. (1997) used X-ray and proton magnetic resonance (^1^H NMR) studies to determine the precise conformation of Δ^9^-THC (Rhee et al., 1997). Dewey (1986) identified Δ^9^-THCA-A from *Cannabis* extract, which is photosensitive and cannot form crystals (structure as compound 2 shown in Table 1) (Dewey, 1986). Devane et al. (1988) discovered Δ^9^-THCA-B (compound 3 in Table 1) from *Cannabis*. *Cannabis* sole, a flat form of illicit *Cannabis*, was eluted from the silicic acid matrix using a 1:1 diethyl ether/petroleum ether solution. Δ^9^-THCA-B was shown to be more polar than Δ^9^-THCA-A in thin-layer chromatography (TLC). The determination of the crystalline structure of Δ^9^-THCA-B was due to the differences in biochemical properties between Δ^9^-THCA-B and Δ^9^-THCA-A (Galal et al., 2009).

Romano and Hazekamp (2019) isolated Δ^9^-tetrahydrocannabivarin (Δ^9^-THCV) using a mixture of 5 g of *Cannabis* and 200 mL of petroleum ether and dissolved it in 100 mL of absolute ethyl alcohol (EtOH) (Romano and Hazekamp, 2019). Spectroscopic evidence for Δ^9^-trans-tetrahydrocannabidiolic acid (Δ^9^-THCVA) was reported by Matsuda et al. (1990), followed by mass spectrometric evidence data (Pate, 1994). The analysis of 51 samples sourced from various geographic regions led to research on the C3 homologs of *Cannabis* (Turner et al., 1973). Balcke et al. (2014) discovered a new homologue of Δ^9^-THC with a methyl side chain, 9-tetrahydrocannabiorcol (Δ^9^-THC-C1), in an extract of Brazilian *Cannabis* (Balcke et al., 2014). The concentration of Δ^9^-THC-C1 was low, so it was not expected to have a significant impact on the drug’s biological action. Dewey (1986) identified Δ^9^-trans-THCA-C4 and Δ^9^-trans-THC-C4 using GC-MS, as well as Δ^9^-trans-tetrahydrocannabiorcolic acid (Δ^9^-THCA-C1) (Balcke et al., 2014). Several techniques, including NMR spectroscopy and Gas Chromatography-Mass Spectrometry (GC-MS), were used to identify monoterpene or sesquiterpene esters of 9-tetrahydrocannabinolic acid A in *Cannabis* sativa L. These esters were found to be precursors to Δ^9^-THC and were broken down into their constituents when subjected to high temperatures during GC-MS analysis (Caspi et al., 2005). Chromatographic methods, such as vacuum liquid chromatography (VLC), High-performance liquid chromatography (HPLC), and Supercritical fluid chromatography (SPC) were used to isolate these cannabinoid esters from high-potency *C. sativa* varieties. *Cannabis*ol, a dimeric cannabinoid, was also isolated using flash silica gel column chromatography from *Cannabis* samples that contained a significant amount of CBG (Costa et al., 2007). Eight new substances of the tetrahydrocannabinol family are listed in Table 2.

**TABLE 2:** Novel substances of the tetrahydrocannabinol class.

S/N	Tetrahydrocannabinol	Molecular structure	Reference
1	β-Fenchyl-Δ^9^-tetrahydrocannabinolate	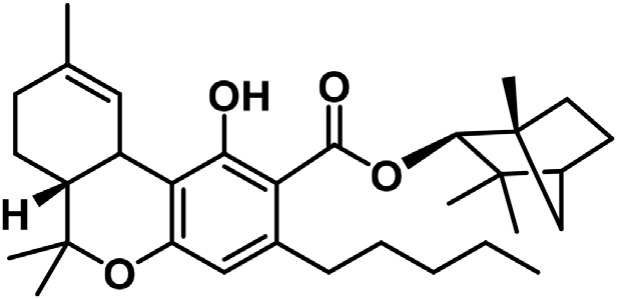	Ahmed et al. (2008)
2	α-Fenchyl-Δ^9^-tetrahydrocannabinolate	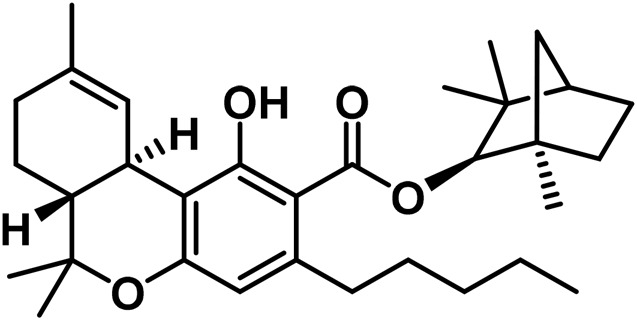	Radwan et al. (2017)
3	*epi*-Bornyl-Δ^9^-tetrahydrocannabinolate	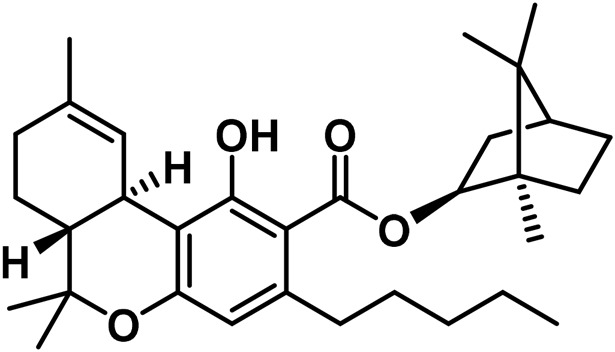	Radwan et al. (2017)
4	Bornyl-Δ^9^-tetrahydrocannabinolate	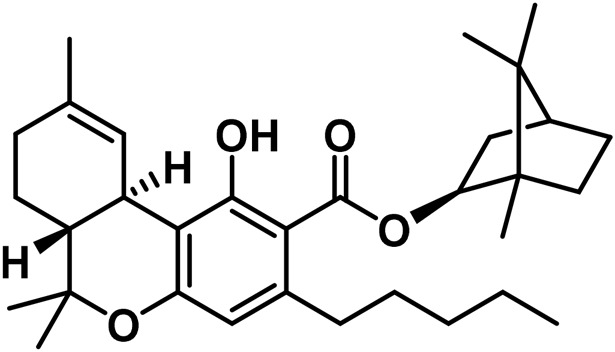	ElSohly et al. (2017)
5	α-Terpenyl-Δ^9^-tetrahydrocannabinolate	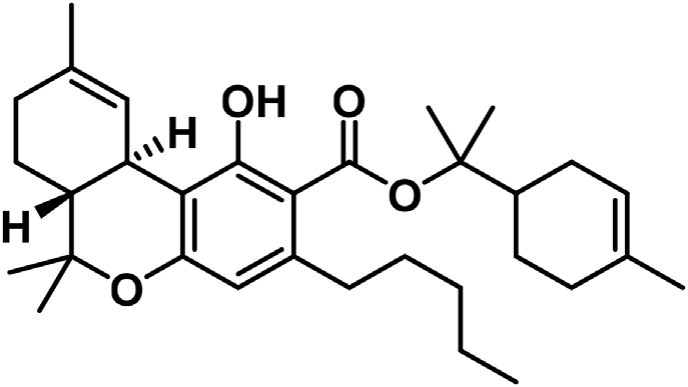	ElSohly et al. (2017)
6	4-Terpenyl-Δ^9^-tetrahydrocannabinolate	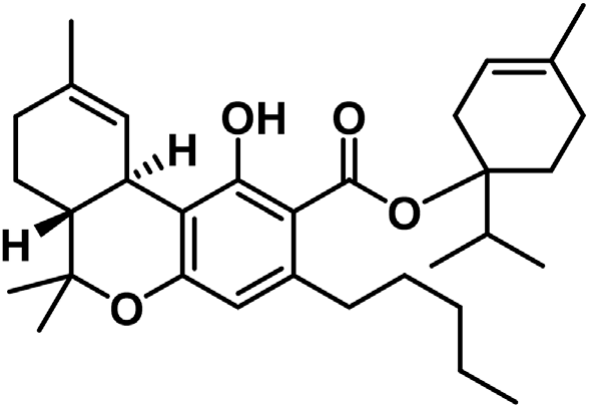	Odieka et al. (2022)
7	α-Cadinyl-Δ^9^-tetrahydrocannabinolate	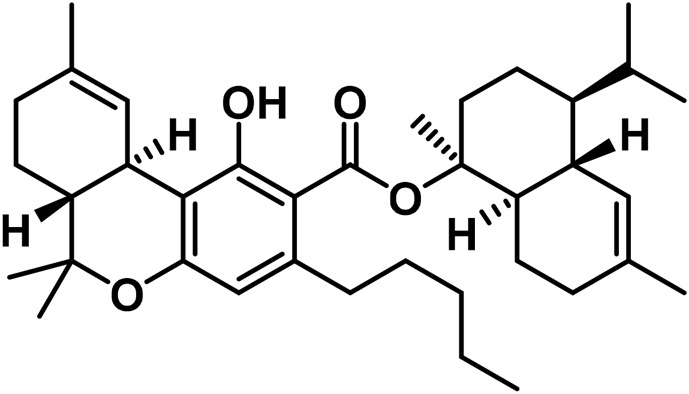	Ahmed et al. (2008)
8	γ-Eudesmyl-Δ^9^-tetrahydrocannabinolate	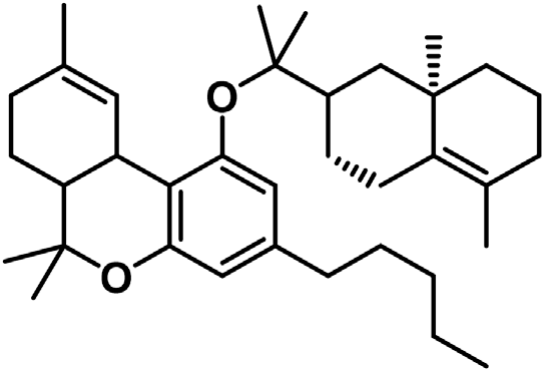	Ahmed et al. (2008)

#### 3.1.2 Cannabigerol (CBG) type

Cannabigerol (CBG) is the first substance purified from *Cannabis* resin (CBG-C5, compound 5 in Table 1) (Mechoulam and Shvo, 1963). Mechoulam et al. (1995) were the first to describe the condensation of geranyl pyrophosphate in the formation of CBG. Mechoulam et al. (1995) discovered that cannabidiolic acid (CBGA) was the most polar acid component. They also found the methyl ester of CBGA in the acidic part of a single extract of *Cannabis* (Mechoulam et al., 1995).

Cannabigerovarinic acid (CBGVA–the structure of compound 1 in Table 3) isolated from an extract of the dried leaves of Thai *Cannabis* was found to be a minor component of the extract (Thomas, 1996; Van Os et al., 2002). After extraction of the acid fraction from the leaves using silica gel column chromatography, the acid fraction was eluted from the dried leaves using a mixture of hexane, ethyl acetate, and a ratio of 5:1 of benzene to acetone. The transparent needle-like CBGVA crystals were obtained following recrystallization in hexane: ethyl acetate solution in a ratio of 3:1.

**TABLE 3:** Summary of some isolated cannabinoids.

No	Cannabinoids	Molecular structure	Part of plant	Extraction solvent	References
1	Cannabigerovarinic acid (CBGVA)	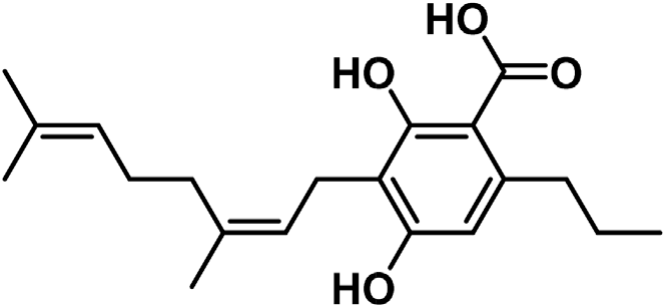	leaves	Benzene, acetone	Thomas (1996), Van Os et al. (2002)
2	γ-Eudesmyl cannabigerolate	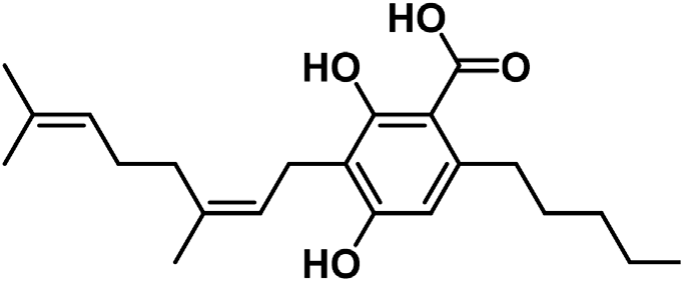	leaves	hexane	Aizpurua-Olaizola et al. (2016)
3	α-Cadinyl cannabigerolate	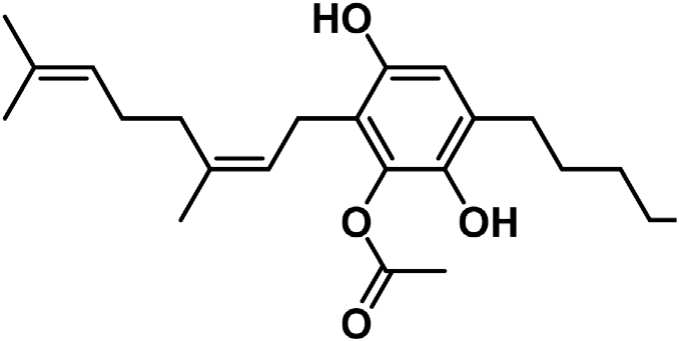	leaves	hexane	Ahmed et al. (2008)
4	Cannabichrome Varinic	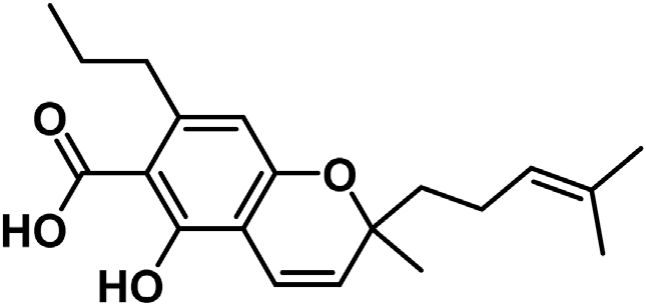	Young leaves	acetone	Showalter et al. (1996)
5	(±)-4-Acetoxycannbichromene	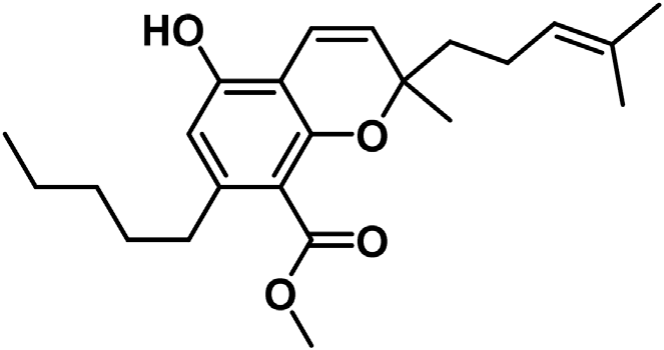	leaves	hexane	Jagannathan (2020)
6	(±)-3′′-Hydroxy- Δ^4^′′-cannabichromene	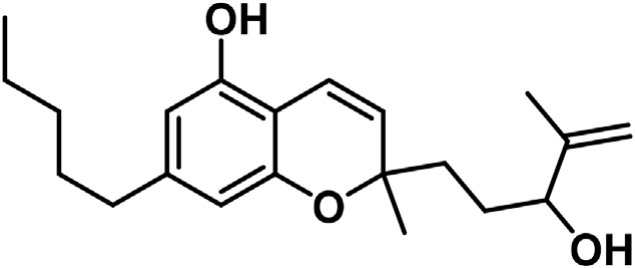	leaves	hexane	ElSohly et al. (2017)
7	(±)-7-Hydroxycannbichromeme	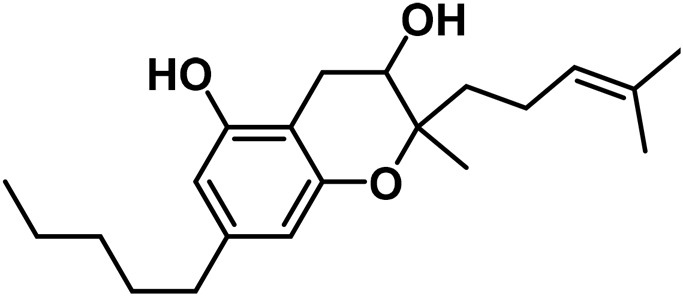	leaves	hexane	Harvey et al. (1977)
8	Cannabinol (CBN)	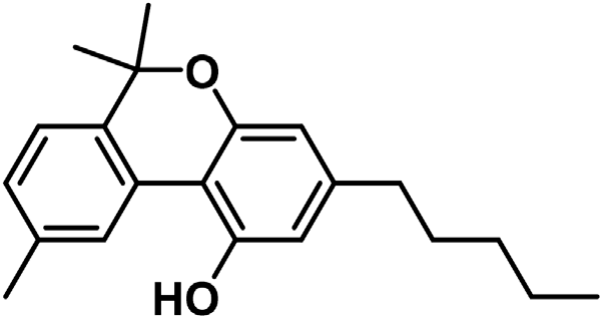	Cannabis resin	ethanol	Wood et al. (1899)
9	Cannabimovone (CBM)	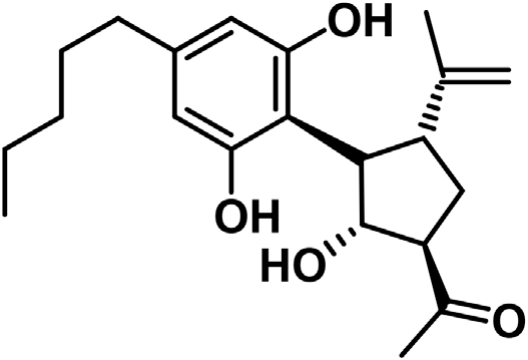	Hemp leaves	hexane	Iannotti et al. (2020)
10	Cannabivarin (CBN-C3)	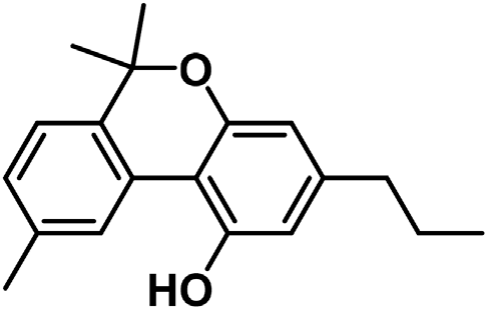	Trichomes of flowers	Chloroform and hexane	ElSohly and Gul (2014)
11	Cannabicyclol (CBL)	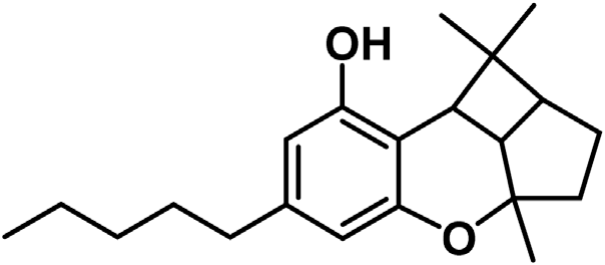	Dried leaves	benzene	Carlini et al. (1974)
12	Cannabicyclolic acid (CBLA)	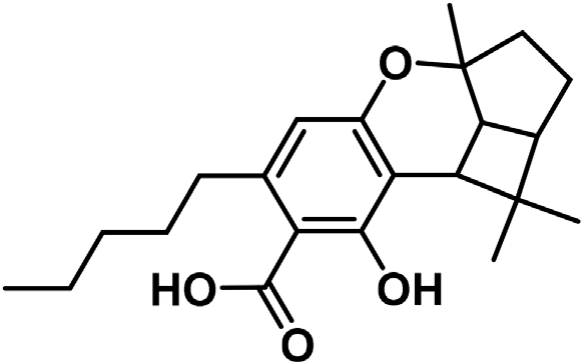	Dried leaves	benzene	Carlini et al. (1974)
13	Cannabicyclovarin	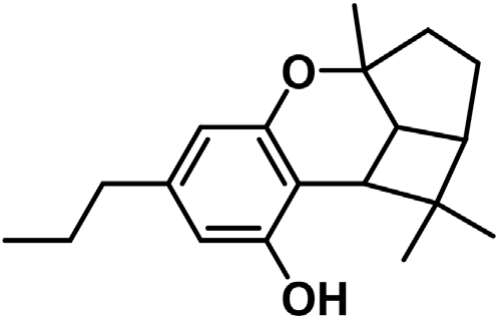	Dried leaves	benzene	Carlini et al. (1974)

Cannabinerolic acid (CBRA) and cannabigerolic acid (CBGA) are both acidic cannabinoids that are produced in the *Cannabis* plant. The primary difference between the two is the location of the double bond in their molecular structures (Taura et al., 1996). CBGA is the precursor to many of the other cannabinoids found in *Cannabis*, including THC and CBD. It is synthesized by the plant from olivetolic acid and geranyl pyrophosphate. CBGA can be further converted into THCA, CBDA, or CBCA, which are then decarboxylated to produce THC, CBD, or CBC (cannabichromene) (Morimoto et al., 1998). Taura et al. (1995) described a procedure to purify cannabinerolic acid from an air-dried Mexican strain of *C. sativa* by extracting the leaves with benzene. The extraction was concentrated and loaded onto a silica gel column, then extracted with a 9:1 (v/v) benzene/acetone mixture after dissolving the residue in acetone and removing any insoluble particulates. High-potency cannabigerolic acid esters, i.e., γ-eudesmyl cannabigerolate and α-cadinyl cannabigerolate were also recovered from *C. sativa* in another study (Kinghorn et al., 2017). The hexane extract of *Cannabis* was purified by chromatography to obtain the two cannabigerolic acid esters. Both γ-eudesmyl cannabigerolate and α-cadinyl cannabigerolate were shown to be esters of CBGA by the data obtained from their respective spectroscopic analyses (Wallace et al., 2001). van Winkel (2011) identified six substances using flash silica gel analysis of a hexane extract, including 5-acetyl-4-hydroxycannabigerol, 4-acetoxy-2-geranyl-5-hydroxy-3-n-pentylphenol, (±)-6,7-trans-epoxycannabigerolic acid, (±)-6,7-cis-epoxycannabigerolic acid and (±)-6,7-cis-epoxycannabigerol (Van Waes et al., 2012). Appendino et al. (2008) isolated a novel, polar dihydroxy cannabigerol derivative (carmagerol) from the *Cannabis* leaves. Taylor et al. (2010) identified sesquicannabigerol, a lipophilic analogue of cannabigerol, in the waxy section of the fiber hemp cultivar Carma. Methanolic potassium hydroxide (·KOH) was used to hydrolyze the wax, and it was purified using gravity silica gel column chromatography before being subjected to flash chromatography over neutral alumina (Taylor et al., 2010)*.*

#### 3.1.3 Cannabichromene (CBC) type

Matsuda et al. (1990) reported the independent discovery of cannabichromene (CBC-C5), which is listed as compound 6 in Table 1. Later, CBC-C5 was isolated from dried the leaves at a yield of 1.5% using a method outlined by Mechoulam et al. (1995) and extracted cannabichromenic acid (CBCA) from the benzene percolate. The production of CBCA using a solvent system of 1:1 hexane and ethyl acetate was confirmed using NMR spectroscopy (Borah and Bordoloi, 2020).

The study found that cannabichromenic acid (CBCA) showed similarities to the structure of THCA in its infrared (IR) spectra due to the placement of the carboxyl group and the presence of intermolecular hydrogen bonding. The researchers isolated cannabichromevarin (CBCV), a brownish-red cannabinoid, from neutral cannabinoids obtained from Thai *Cannabis* leaves through multiple passes through a silica gel column and elution with benzene and 20:10:1 benzene-hexane (Showalter et al., 1996). Cannabichromevarinic acid (CBCVA), was isolated in young leaves of *Cannabis*, using acetone (Showalter et al., 1996). Synthesis was used to validate the structure of natural CBCVA. Lakhan and Rowland (2009) reported the isolation of three new cannabichromene type cannabinoids from high-potency benzene extract of the flowers (trichomes). These cannabinoids are named (±)-4-acetoxycannabichromene, (±)-3″-hydroxy-Δ^4^″-cannabichromene and (±)-7-hydroxycannabichromeme (Lakhan and Rowland, 2009).

#### 3.1.4 Cannabidiol (CBD) type

The two main metabolites of non-psychotropic (fiber-type) *Cannabis* cultivars are cannabidiol (CBD) and cannabidiolic acid (CBDA), their structures were shown in Table 1 as compounds 7 and 8, respectively. CBD was isolated, from the ethanol extract of leaves, and after being left for several weeks, the oily CBD was crystallized (Karniol and Carlini, 1973). Li (1974) reported its synthesis and absolute configuration as (−)-*trans*-1R, 6R. Cannabidavarin (CBDV) was isolated from an ethanol extract of *Cannabis* olein (flower), which was chromatographed on silica gel (Iwamura et al., 2001). Howlett et al. (2002) extracted neutral cannabinoids from the ethanol extract of leaves to produce cannabidiol monomethyl ether (CBDM) (M-1). Benzene was used to elute the cannabinoids after they had been chromatographed on Florisil. To produce CBDM, the eluted fraction was rechromatographed on silica gel and eluted with a ratio of 3:1 hexane/benzene.

*Cannabis* resin and leaves that had been crushed were percolated with ethyl acetate to produce a residue that was filtered and concentrated. This residue was derivatized prior to GC-MS analysis. The mass and methylene unit of cannabidiol-C4 allowed for its identification (Harvey, 1976). Hall and Degenhardt (2007) extracted cannabidiolic acid (CBDA) from the benzene extract of Thailand *Cannabis*. Cannabimovone (CBM) is a polar cannabinoid that was isolated from an acetone extract of *Cannabis sativa* L. leaves which is not psychoactive (Hayakawa et al., 2008).

#### 3.1.5 Cannabidiol (CBND) type

**FIGURE 4:**
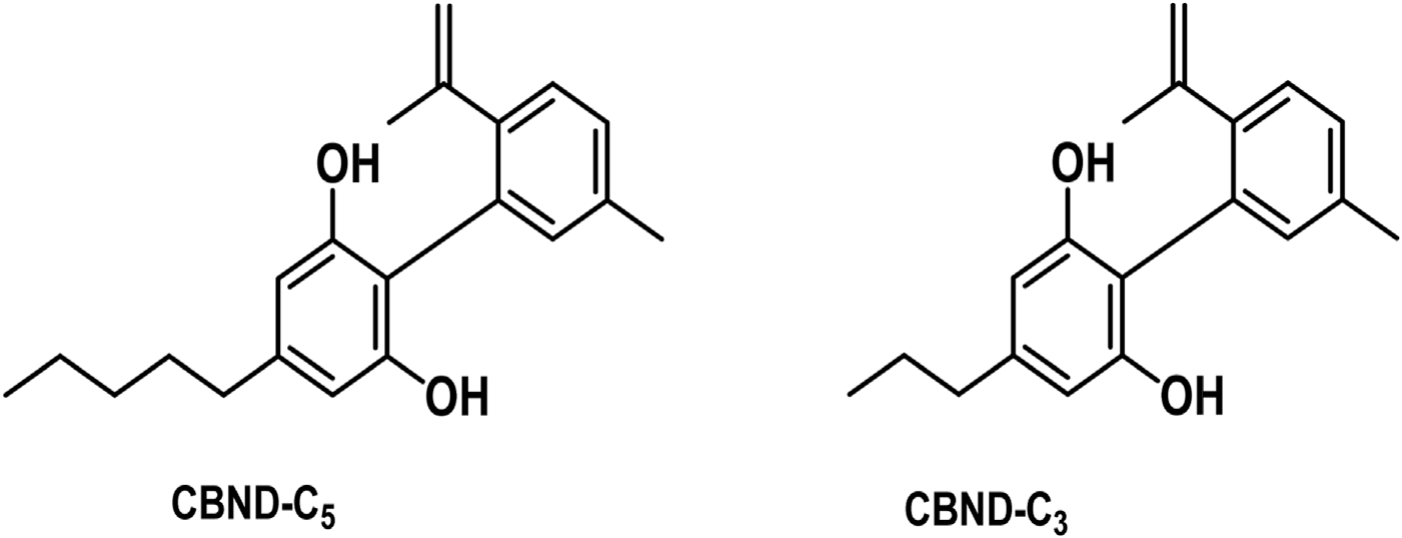
CBND-type cannabinoids.

The aromatized derivatives of CBD are called CBND-type cannabinoids (Figure 4). The two compounds in this subclass that are characterized are cannabidiol (CBND-C_5_) and cannabidiol (CBND-C_3_) (Gaoni and Mechoulam, 1964). By using a hexane-ether extract of Lebanese *Cannabis* (resinous trichomes), Gorelick and Heishman (2006) were able to successfully isolate cannabidiol. GC-MS analysis revealed the presence of cannabidiol-C3, the propyl homolog of cannabidiol-C5 (Bhattacharyya et al., 2010).

#### 3.1.6 Cannabielsoin (CBE) type

The Cannabielsoin (CBE-C5), Cannabielsoic acid A (CBEA-C5 A), Cannabielsoic acid B (CBEA-C5 B), Cannabielsoin-C3 (CBE-C3), and Cannabielsoic-C3 acid B (CBEA-C3 B) are the five cannabielsoin-type cannabinoids present in *Cannabis* (Mechoulam et al., 1995). CBE was isolated from an ethanolic extract of hashish (resinous trichomes of flowers) originating in Lebanon (Di Forti et al., 2009). CBEA-C5 A and CBEA-C5 B were extracted from a benzene extract of *Cannabis* (resinous trichomes) that was grown in Lebanon (Mechoulam and Shvo, 1963).

#### 3.1.7 Cannabicyclol (CBL) type

**FIGURE 5:**
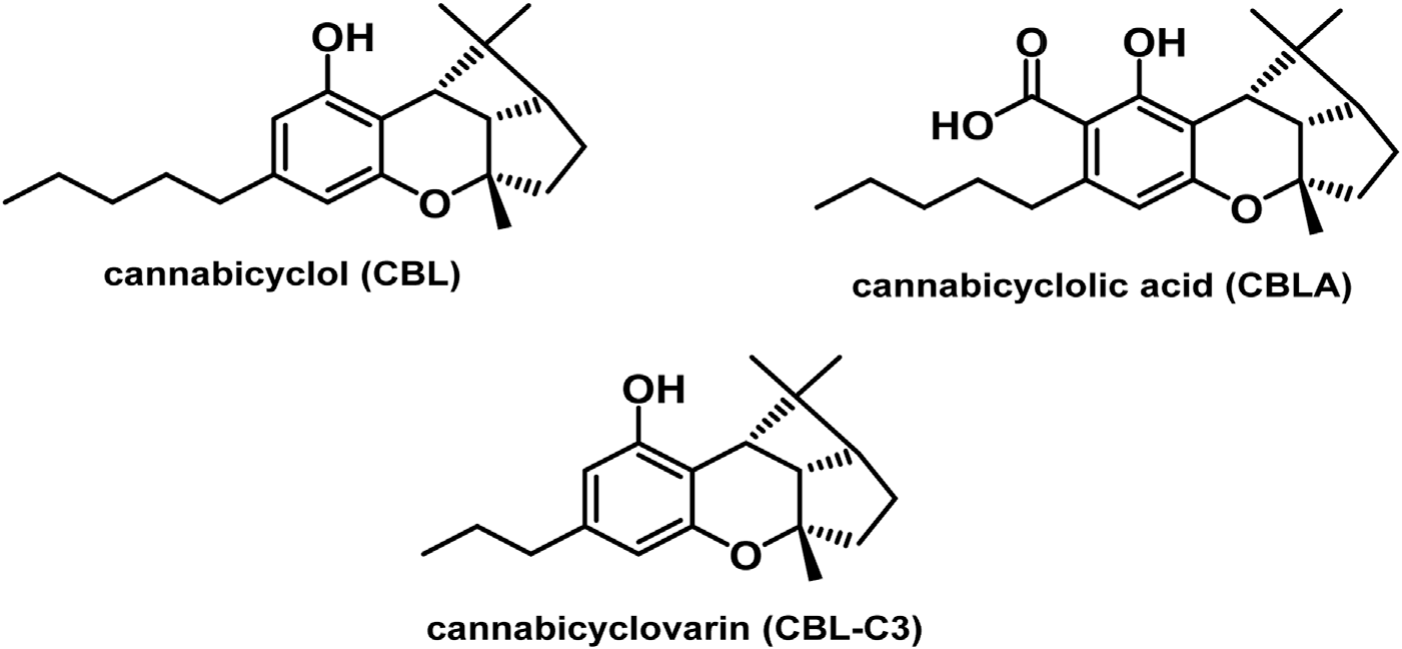
Types of cannabicyclols.

The only compounds that have been identified from this subclass are known as cannabicyclol (CBL), cannabicyclolic acid (CBLA), and cannabicyclovarin (CBL-C3) (Figure 5) (Carlini et al., 1974). Benowitz and Jones (1981) are credited with being the first to identify CBL. They used TLC to isolate CBL from a variety of benzene extract of dried leaves of *Cannabis* samples.

#### 3.1.8 Cannabinol (CBN) type

Cannabinol (CBN) was given its name for the first time in 1896 (Wood et al., 1899). CBN was made into oil by extracting *Cannabis* resin using ethanol and heating it. After some time, the oil was acetylated to get pure CBN in the form of its acetate. Bhattacharyya et al. (2009) were able to correctly estimate the structure of CBN. A crude acidic fraction of hashish was used to isolate cannabinolic acid A (CBNA), which was then esterified with diazomethane and purified as its methyl ester on an acid-washed alumina column (Mechoulam and Shvo, 1963). Cannabivarin (CBN-C3) was extracted using a mixture of chloroform and hexane from Nepalese hashish (resin trichomes of flowers), and the structure of the compound was validated by mass spectrum data (ElSohly and Gul, 2014). A summary of some isolated cannabinoids is presented in Table 3.

### 3.2 Other phytochemicals in *Cannabis*

#### 3.2.1 Terpenes

Terpenes are aromatic compounds that are found in many plants, and they perform various biological roles, such as attracting pollinators and protecting against predation (Tetali, 2019). In the *Cannabis* plant, terpenes are stored as essential oils. Currently, over 200 distinct terpenes have been identified in *Cannabis*, with most of them being discovered through steam distillation (Booth and Bohlmann, 2019).

Terpene concentrations can vary due to various genetic factors. In *Cannabis* flowers, terpenoid concentrations were found to range from 1% up to 10% within the trichomes as of 2009. However, selective breeding has led to an increase in terpenoid concentrations found in flowers in recent years, with some chemovars exhibiting concentrations of 3.5% or higher (Feder et al., 2021). Currently, over 50 different terpenes have been identified in *Cannabis*, with a few dominating compounds classified as the “terpene super class,” including linalool, ocimene, limonene, myrcene, α-pinene, humulene, β-caryophyllene, and terpinolene (Liktor-Busa et al., 2021). Similarly, Fischedick and others (2017) analyzed *Cannabis* samples and classified them into five distinct groups based on the above terpenoid classifications (Fischedick, 2017).

Secondly, several terpenes found in *Cannabis* exist as hydrocarbons which are direct products of terpene synthase enzymes as compared to complex terpenes that require adjustments by other enzymes such as cytochrome P450 (ElSohly, 2002). It can be concluded that the chemical diversity of terpenes in *Cannabis* is a direct reflection of the encoding enzymes in *Cannabis*. Other common terpenes in *Cannabis* are bisabolol, sesquiterpenes, and β-farnesene, (Booth, 2020). Monoterpenes have a ten-carbon isoprenoid precursor known as the geranyl diphosphate (GPP), while sesquiterpenes have a fifteen-carbon isoprenoid farenesyl diphosphate (FPP) (Stasiłowicz et al., 2021). Therefore, in the synthesis of sesquiterpenes and monoterpenes, GPP and FPP act as substrates in producing different structures of terpenes.

#### 3.2.2 Flavonoids

At least 20 flavonoids have been found in *Cannabis*, most of which are flavanols and flavones (Li et al., 2022). In 2011, three geranylated flavones known as cannflavin A, B, and C were found in the plant (Bautista et al., 2021). Currently, the leaves, flowers, seedlings, and fruits of *C. sativa* have been found to contain flavonoids that remain undetected in roots and seeds (Eggers et al., 2019). Apart from finding this compound in specific regions of the plant, flavonoids have been identified to vary in bracts during plant development (Ross et al., 2005).

Since several flavonoids have protective functions, their production is dependent on environmental factors that have been found in several plants as well as *Cannabis*. For instance, the accumulation of cannflavin A is predisposed to genetic variations, as well as environmental factors such as temperature, rainfall, and humidity in the environment (Kumar and Pandey, 2013). Besides, the contents of cannflavin A, B, and C in cloned species of *C. sativa* vary at different altitudes (Wiles et al., 2022). With these findings, it can be postulated that identifying unknown flavonoids in the plant, is reliant on certain environmental conditions or stresses. Another study by Pavlovic et al. (2019) confirms that certain flavonoids are produced in significant quantities in hexane extracts of flowers of *C. sativa* chemovars like cannflavcin C. Thus, identifying more flavonoids in *C. sativa* will provide a comprehensive understanding of its biosynthesis and functions in the plant.

*Cannabis* has 26 distinct flavonoids (ElSohly, 2007). There are various flavonoids in *Cannabis*, but the most important ones are orientin, vitexin, luteolin-7-O-glucoside, and apigenin-7-O-glucoside (ElSohly, 2007). Moreover, it contains the potent antioxidant, quercetin (Mnekin and Ripoll, 2021). Cannabinoids are a new type of flavonoid. They are made up of three chemicals found only in *Cannabis*, i.e., Cannflavin A, B, and C. Cannflavins were discovered in hemp’s leaves and blooms (Werz et al., 2014). Cannflavin A is 30 times more anti-inflammatory than aspirin (Barrett et al., 1985). This anti-inflammatory activity can be explained by the reduction of mPGES-1 and 5-LO (Erridge et al., 2020).

#### 3.2.3 Steroids

Presently, steroid compounds such as campesterol, sitosterol, and stigmasterol have been identified in *Cannabis* roots (Ryz et al., 2017). Moreover, eleven phytosterols have been found in the plant which belongs to the groups stated above (Farinon et al., 2020). A trifecta of sterols (campesterol, stigmasterol, and sitosterol) was extracted using hexane from the seed oil of the Indian *Cannabis* strain (Jurgoński et al., 2020). These three phytosterols were also found in *Cannabis* smoke, according to research by Kumar et al. (2021). Furthermore, β-sitosterol-3-O—Dglucopyranosyl-60-acetate, a known sterol, was first isolated from roots, stem bark, and leaves of *Cannabis* by using a mixture of methanol and chloroform solvent in the ratio 9:1 (Jin et al., 2020). Sitosterol and sitosterol-D-glucoside were extracted from the plant’s roots in the same study using a mixture of methanol and chloroform solvent in a ratio of 9:1 (Jin et al., 2020). Recent research by Ferrini et al. (2021), found that higher concentrations of campesterol, stigmasterol, and sitosterol were associated with higher total sterol levels in flowers, leaves, roots, and stems (Ferrini et al., 2021).

#### 3.2.4 Alkaloids

Alkaloids are part of the chemical defense mechanism used by plants to ward off herbivores (Walters, 2011). It has been shown that *Cannabis* contains endogenous indole alkaloids (Fasakin et al., 2022). For example, alkaloids may be used as analgesics, antibiotics, anticancer drugs, antiarrhythmics, asthma medications, antimalarials, anticholinergics, bronchodilators, laxatives, miotics, oxytocics, vasodilators, psychotropics, and stimulants (Manske and Holmes, 2014). Included in this group of chemicals are morphine, cocaine, nicotine, caffeine, quinine, ephedrine, and many more (Yan et al., 2016).

A group of researchers led by Klein in 1971 researched *Cannabis* alkaloid combinations and reported the isolation of four different alkaloids, which they called cannabimines A-D (Garcia-Romeu et al., 2016). In the study of cannabinoids, *Cannabisativine* was the pioneering alkaloid. In 1975, it was extracted from the roots of a Mexican variety of *Cannabis* sativa that was growing in Mississippi, United States. The compound was extracted using methanol as a solvent. (Chandra et al., 2017). These alkaloids were shown to have diuretic, analgesic, anticancer, antipyretic, and antiemetic effects (Lata et al., 2016).

In 1881, Siebold and Bradbury presented their findings on the separation of the alkaloid cannabinine at the British Pharmaceutical Conference (Warden, 1885). The next year, in 1883, Hay discovered tetanocannabin, another physiologically active alkaloid. Due to its ability to induce convulsions in amphibians similar to those caused by strychnine, the compound earned its name, a “cannabine alkaloid” product, that was marketed by Merck (of Darmstadt) as early as 1986 (Lowe et al., 2021).

#### 3.2.5 Fatty acids

Fatty acids carry out their physiological activities due to the involvement of their functional groups in various chemical processes. Some of the fatty acids produced by *Cannabis* sativa L. can be identified based on their chemical structure (Mölleken and Theimer, 1997). The fatty acids produced by *Cannabis* sativa L. have a specific chemical structure that can be distinguished from other fatty acids based on their unique features (Babiker et al., 2021). In 1996, Ross and others investigated the fatty acid profile of lipid matter in commercialized *Cannabis* seeds from several geographical locations. Omega-3 fatty acids such as linolenic acid, isolinolenic acid, and eicosapentaenoic acid; omega-6 fatty acids such as linoleic acid and others such as Caproic acid, caprylic acid, myristic acid, palmitoleic acid, palmitic acid, margaric acid, oleic acid, stearic acid, arachidic acid, isoarachidic acid, and behenic acid are just some of the fatty acids found in commercial *Cannabis sativa* (Kriese et al., 2004). The oil content of the *Cannabis* plant varies depending on various factors such as the cultivar, growing conditions, and the part of the plant that is being analyzed. In general, the oil content of the seeds of the *Cannabis* sativa plant is typically around 30%–35% on a dry weight basis (Mihoc et al., 2012). However, the oil content of other parts of the *Cannabis* plant, such as the flowers or leaves, is generally much lower (8%–16%) than that of the seeds (Novak et al., 2001). Therefore, the seeds are the primary source of oil extracted from the *Cannabis* plant for both industrial and nutritional purposes (Potter, 2014). *Cannabis* is not typically considered a significant dietary source of fatty acids. While *Cannabis* does contain various fatty acids, the concentrations are relatively low compared to other food sources (vegetable oils) that have high concentrations of fatty acids (Callaway, 2004). Almost half of hulled *Cannabis* seeds are made up of fat (triglycerides), and the oil that is extracted from them is unique among cooking oils because its triglycerides include very low levels of saturated fatty acids (0.9%) and extremely high levels of polyunsaturated fatty acids (80%) (Rasool, 2018; Krist, 2020). Linoleic acid (18:2ω6, 54%–60%), α-linolenic acid (18:3ω3, 18%–23%), and oleic acid (18:1ω9, 7%–12%) are the three primary fatty acids found in *Cannabis* seed oil.”

Additionally, *C. sativa* L. seeds, offer nutritional value, since they are composed of around 25% highly nutritious protein and 35% fat (Farinon et al., 2020). Hemp oil, pressed from the seeds of the *Cannabis* plant, is rich in a wide variety of nutrients, including essential fatty acids (EFAs) such as omega-3 and omega-6 fatty acids, vitamins (C, E, B1, B2, B6, B12, and folate), minerals (including calcium, magnesium, potassium, phosphorus, iron, zinc, sodium, and copper), and macronutrients (fat, carbohydrates, fiber, and protein) (Silver et al., 2021).

#### 3.2.6 Waxes

Plants create waxes, a class of non-volatile, larger molecular weight, hydrophobic chemicals, to shield their leaves and stem from dehydration and disease (Ribeiro et al., 2021). They may also serve to stabilize defense chemicals, like the phytocannabinoids and terpenes found in *Cannabis*, which are produced on plant inflorescence (flower heads) (Romero et al., 2020). If compared to *Cannabis* leaves, the wax content in *Cannabis* inflorescence is three times higher (Tipple et al., 2016). The initial stage in the creation of therapeutic *Cannabis* products is commonly the extraction of *Cannabis* inflorescence using organic solvents or supercritical Carbon dioxide (CO_2_), often resulting in a ‘resin’ with a high wax concentration (Ramirez et al., 2019; Sawicka et al., 2021). As a result, waxes are a crucial category of phytochemicals to consider during such production. N-pentacosane (C_25_H_52_), n-heptacosane (C_27_H_56_), n-nonacosane (C_29_H_60_), and n-hentriacontane (C_31_H_64_) are the most prevalent straight-chain hydrocarbons in *Cannabis* waxes (Adams and Jones, 1973). A summary of the structures of other important phytochemicals are provided in (Table 4).

**TABLE 4:** Structure of other phytochemical classes found in the *Cannabis* plant.

No	Phytochemicals	Chemical formula	Molecular structure	Reference
A	Terpenes			
1	Myrcene	C_10_H_16_	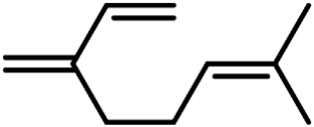	Liktor-Busa et al. (2021)
2	α-Ocimene	C_10_H_16_	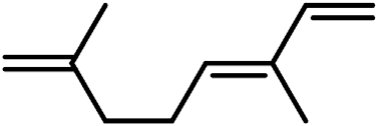	ElSohly et al. (2017)
3	cis-β-Ocimene	C_10_H_16_	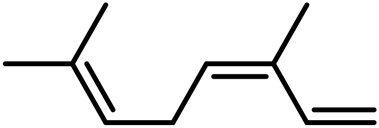	Liktor-Busa et al. (2021)
4	D-Limonene	C_10_H_16_	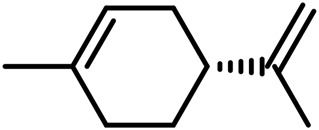	Liktor-Busa et al. (2021)
5	α-Terpinene	C_10_H_16_	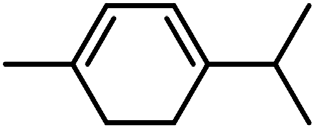	Tomko et al. (2020)
6	Terpinolene	C_10_H_16_	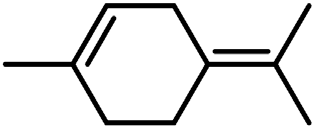	Fischedick (2017)
7	α-Pinene	C_10_H_16_	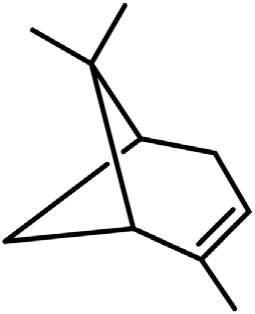	Tomko et al. (2020)
8	Linalool	C_10_H_18_O	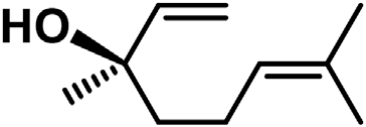	Fischedick (2017)
	Sesquiterpenes			
9	Humulene	C_15_H_24_	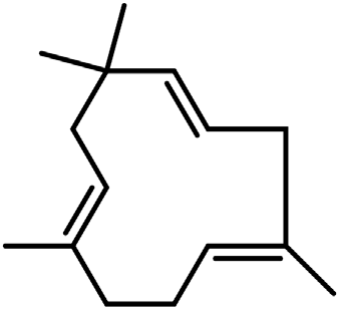	Sommano et al. (2020)
10	α-Farnesene	C_15_H_24_	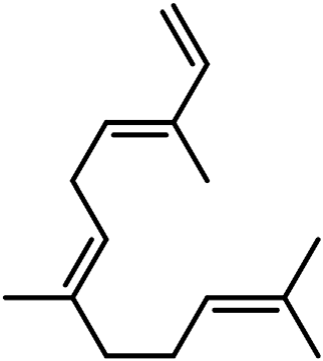	Sommano et al. (2020)
11	β-Farnesene	C_15_H_24_	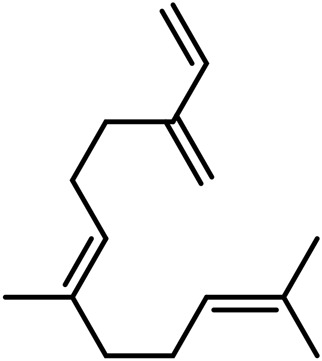	Radwan et al. (2021)
12	β-Caryophyllene	C_15_H_24_	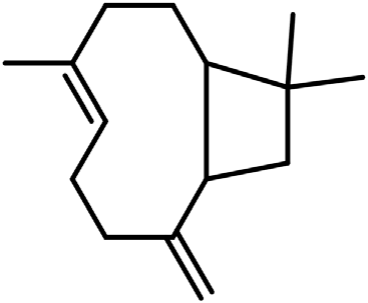	Booth and Bohlmann (2019)
13	α-Bisabolol	C_15_H_26_O	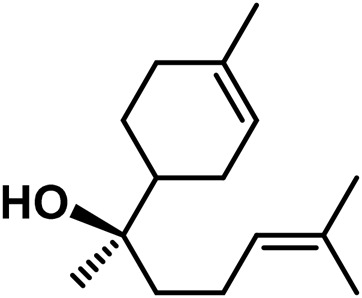	Radwan et al. (2021)
B	Flavonoids			
14	Apigenin	C_15_H_10_O_5_	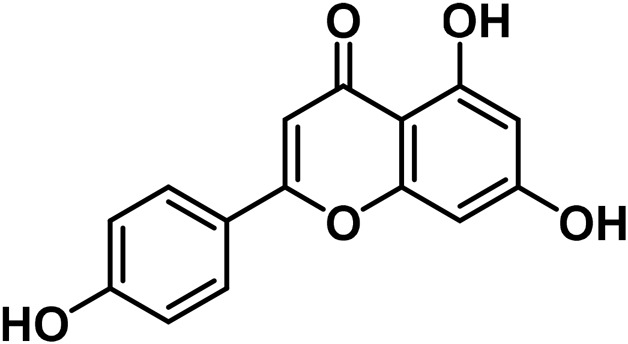	Li et al. (2022)
15	Cannflavin A	C_26_H_28_O_6_	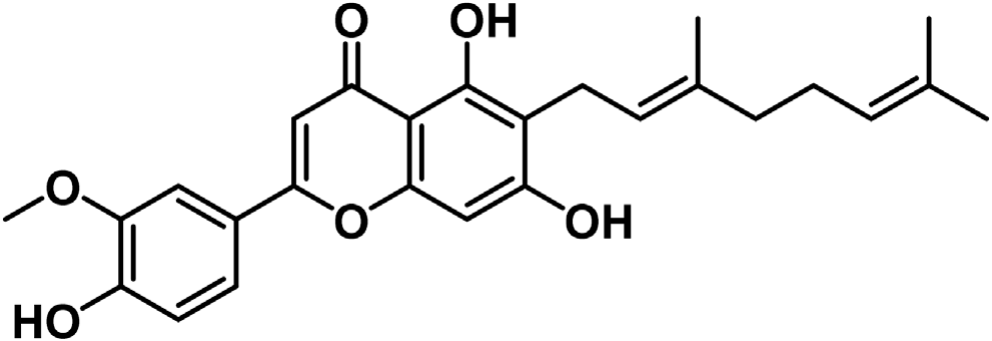	Pollastro et al. (2018)
16	Cannflavin B	C_21_H_20_O_6_	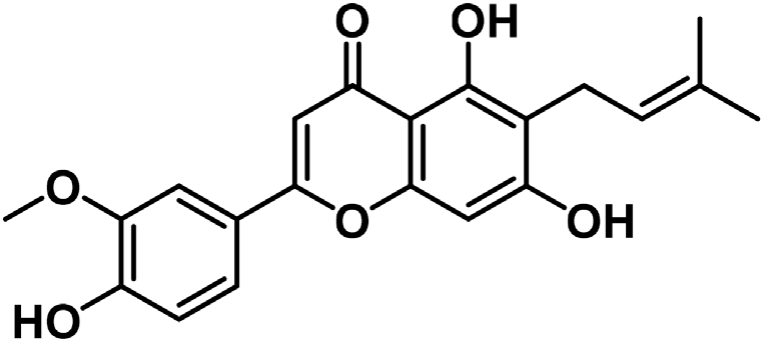	Wiles et al. (2022)
17	Cannflavin C	C_26_H_28_O_6_	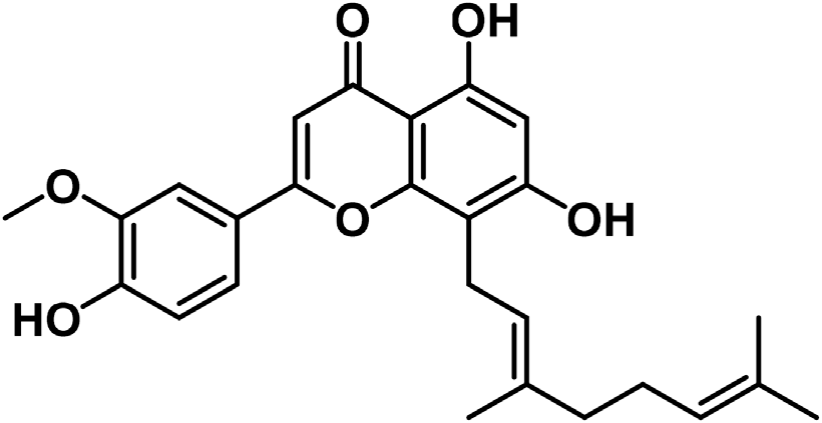	Wiles et al. (2022)
18	Luteolin	C_15_H_10_O_6_	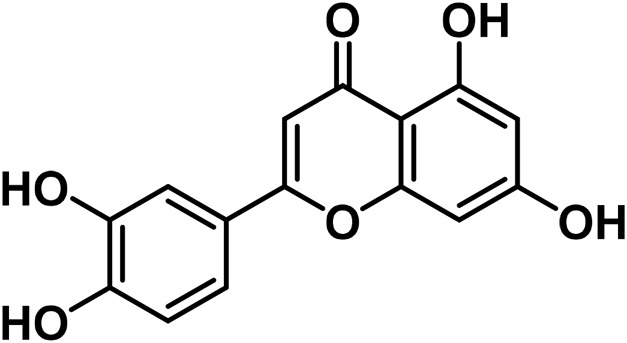	Clark and Bohm (1979)
19	Orientin	C_21_H_20_O_11_	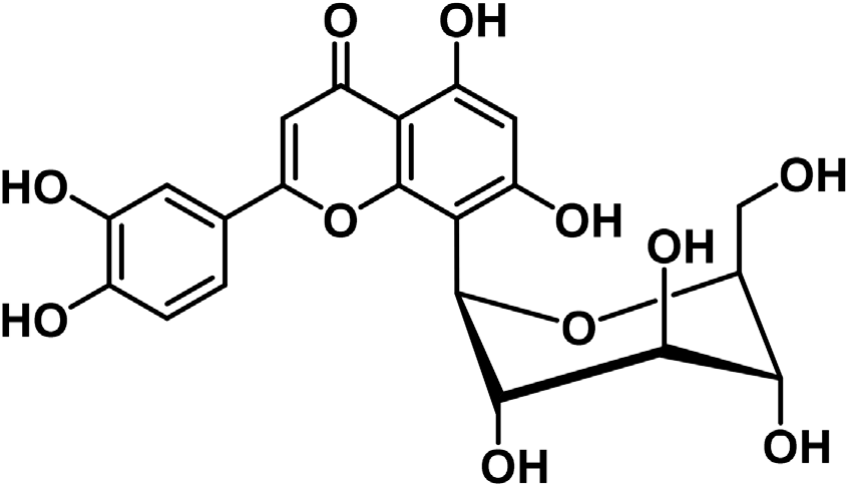	Clark and Bohm (1979)
20	Vitexin		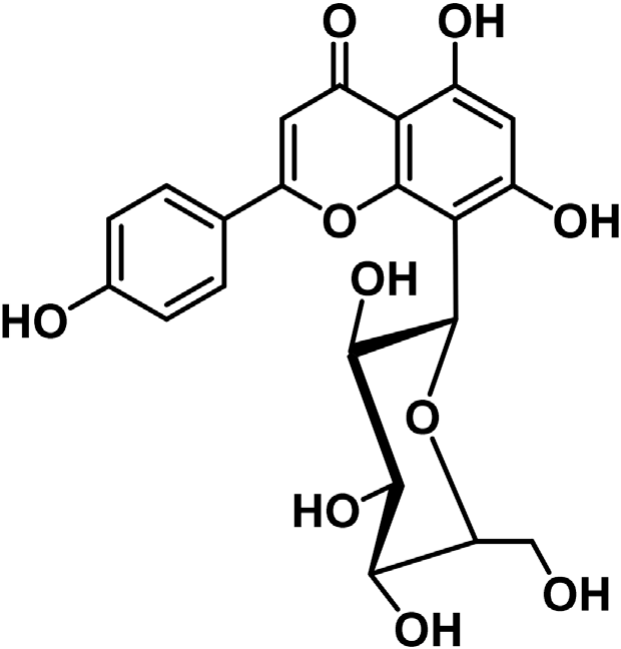	Clark and Bohm (1979)
C	Steroids			
21	Campesterol	C_28_H_46_O	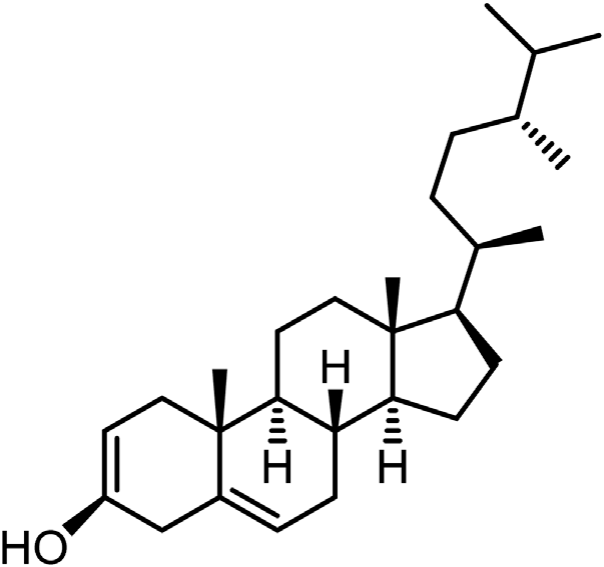	Ryz et al. (2017)
22	β-Sitosterol	C_29_H_50_O	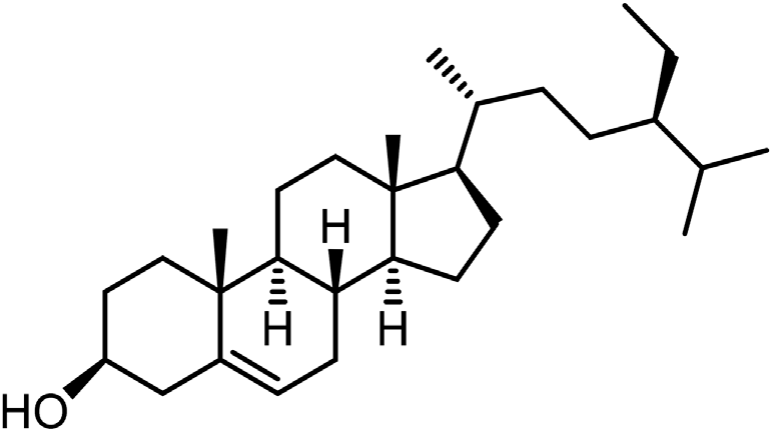	Ryz et al. (2017)
23	Stigmasterol	C_29_H_48_O	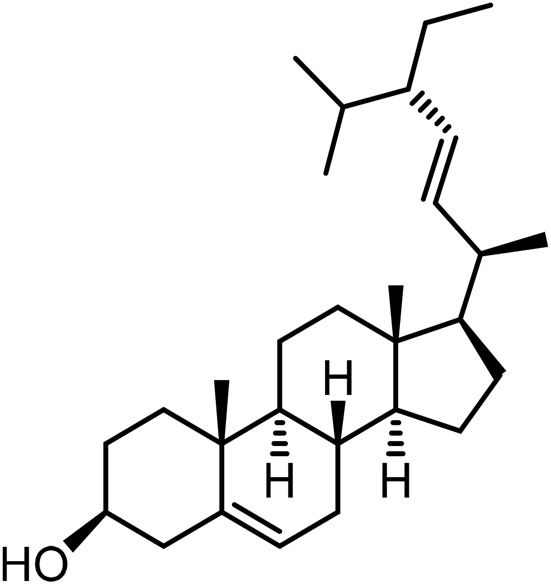	Ryz et al. (2017)
D	Alkaloids			
24	Cannabimines A	C_21_H_37_N_3_O_2_	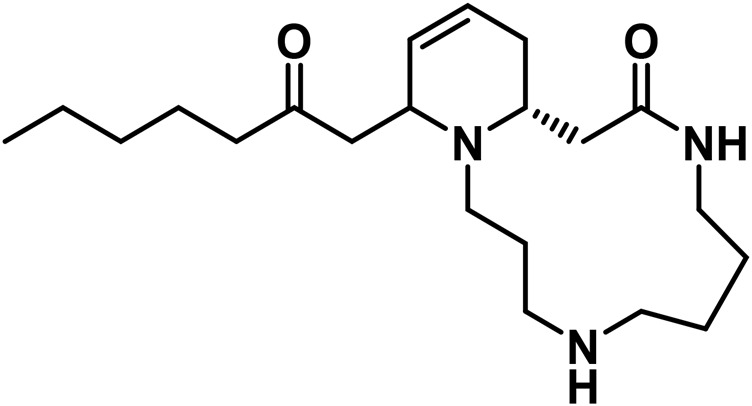	Mechoulam and Hanuš (2000)
25	Cannabisativine	C_21_H_39_N_3_O_3_	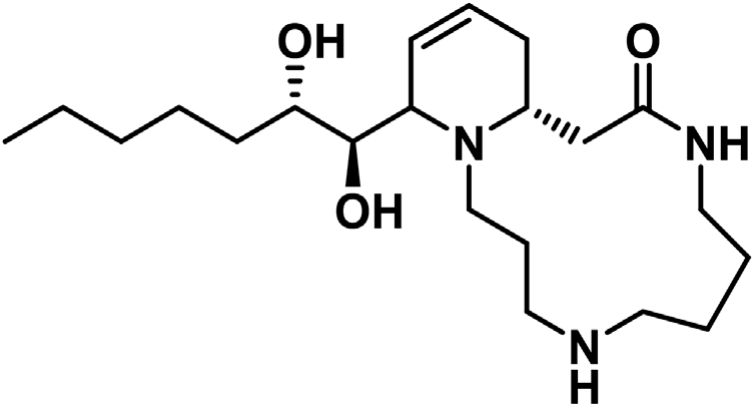	Mechoulam and Hanuš (2000)
E	Fatty Acids			
26	Caproic acid	C_6_H_12_O_2_	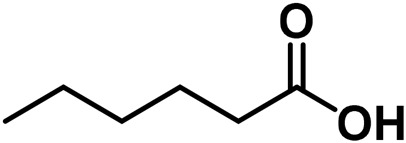	Ross et al. (1996)
27	Eicosapentaenoic acid	C_20_H_30_O_2_	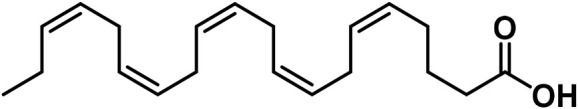	Mölleken and Theimer (1997)
28	Linoleic acid	C_18_H_32_O_2_	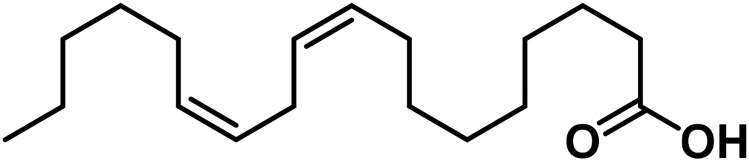	Ross et al. (1996)
29	α-Linolenic acid	C_18_H_30_O_2_	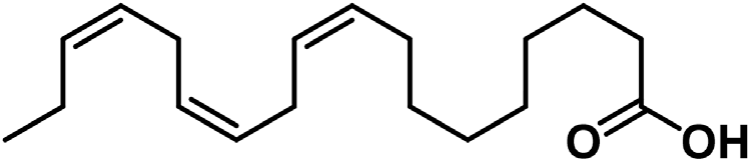	Mölleken and Theimer (1997)
30	γ-Linolenic acid	C_18_H_30_O_2_	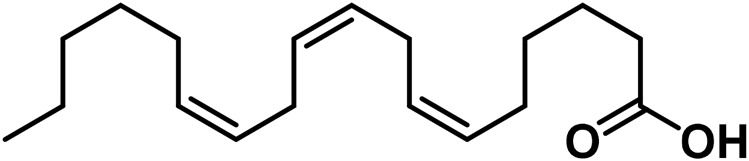	Ross et al. (1996)

## 4 Traditional and psychedelic properties of *Cannabis*

The *Cannabis* plant has been used for centuries and is one of the most beneficial plant genera. Historically, the seeds were used for making oil and pickles, while the leaves were the second most consumed part of the plant and were used in various ways, such as seasoning, flour, and added to meals (Balant et al., 2021). Historically, the psychedelic and recreational use of *Cannabis* dates to the early 1800 s in tropical parts of the world such as South America and Africa. However, the psychedelic use of the plant did not make it to Europe and America until after the 1800 s (Balant et al., 2021). In countries outside the tropics, the psychoactive components of *Cannabis* are not present in the variants grown. *Cannabis* has a long history of medicinal and psychoactive use in India, and it became known in America and Europe in the 19th century for its narcotic and stimulant properties (Tipparat et al., 2012).

*Cannabis* cultivation, commercialization, and use as a recreational drug has a significant incidence on a global basis, and often fall within the realm of illicit activities (Carnevale et al., 2017). However, the comparatively low proportion of psychotropic use does not match the significance of these activities (Carnevale et al., 2017). The CANNUSE database documents various methods of using *Cannabis* for psychoactive purposes, including smoking leaves or inflorescences, ingesting preparations made from leaves, inflorescences, and shoots, and consuming preparations of varying intensity such as charas, attar, hashish, ganja, and plant powder (Boniotti and Griffith, 2002; Balant et al., 2021).

The most common ways of administering *Cannabis* for psychoactive purposes are smoking (56.6%), drinking (37.74%), and eating (5.66%) (Balant et al., 2021). The leaf is the most commonly used part of the plant, accounting for 46.44% of use, even though inflorescences have the highest concentration of THC and other cannabinoids (Hasan, 1975).

The *Cannabis*-based food industry mostly uses seeds and derivatives, but other plant parts like sprouts, leaves, and flowers are consumed raw in dishes and drinks. These plant parts contain higher levels of bioactive phytochemicals like polyphenols and cannabinoids than seeds. Ingesting *Cannabis* makes up 7.29% of all uses, with 58.72% corresponding to traditional meals and 41.28% to traditional beverages. Seeds are the most common plant component used for food, and this association is statistically significant. Seeds are popular among Asian senior citizens due to their high protein content and low glycemic index (Haldar et al., 2019). *Cannabis* seeds can be found in different forms such as energy bars, chocolates, flour, baked products, milk, and flavoring sauce. The plant’s sprouts, leaves, and flowers are also consumed raw in dishes and drinks (Iftikhar et al., 2021; Kuppuram, 2022; Xu et al., 2022).

## 5 Pharmacological potential of *Cannabis*

**FIGURE 6:**
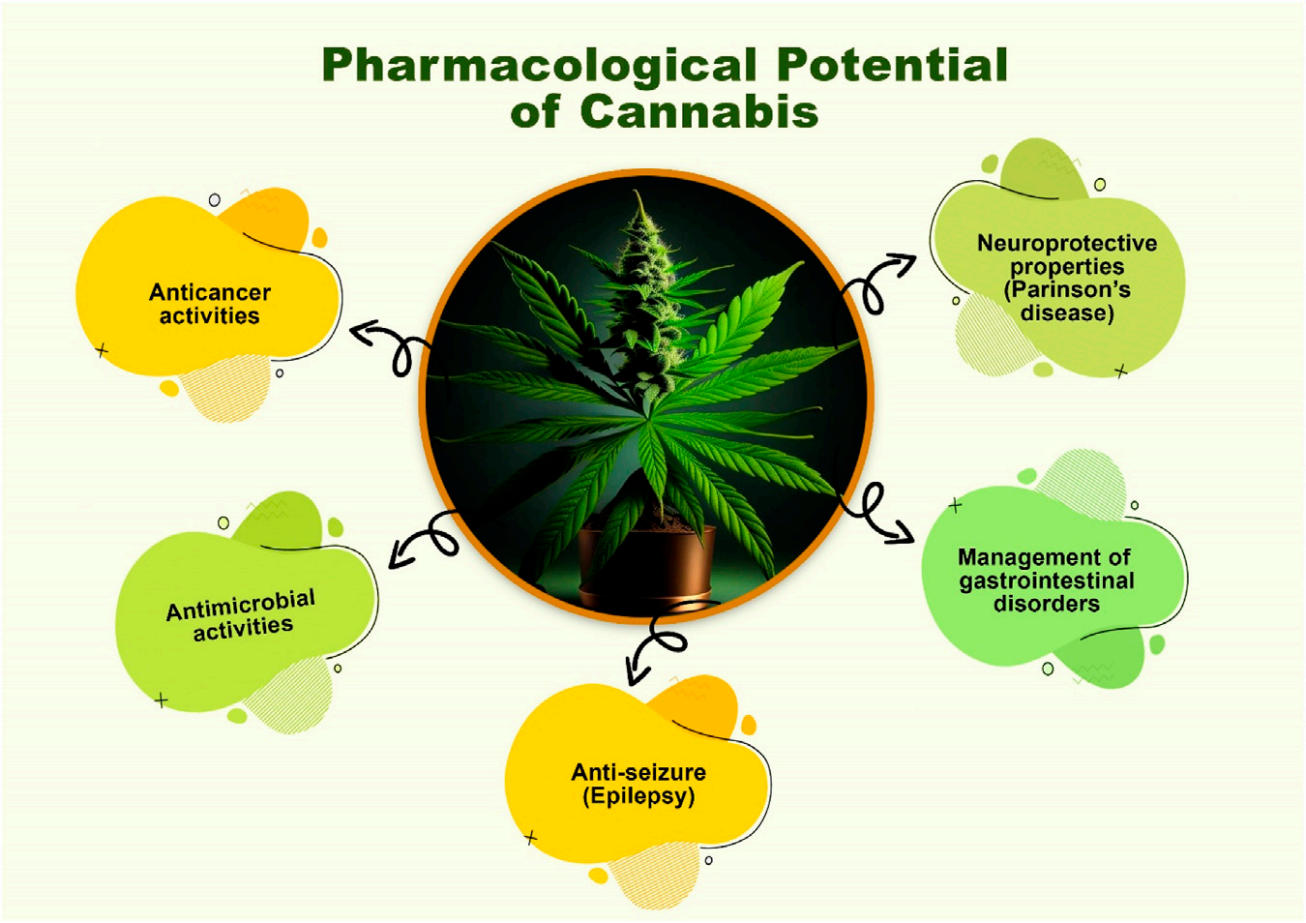
Pharmacological potential of *Cannabis*.

The prospect of using *Cannabis* for the treatment of a wide variety of diseases is promising now that the Δ^9^-THC and endocannabinoid systems, receptors, enzymatic systems, and physiological effects have been identified (Maroon and Bost, 2018). It has been effective in the treatment of gastrointestinal disorders, infections, psychosis, anxiety, depression, anorexia, and cachexia, as well as in the treatment of asthma (bronchiectasis), pain, musculoskeletal disorders, tumor, and arthritis (Śmiarowska et al., 2022). It also has antiglaucoma, antimicrobial, and antiemetic properties. Additionally, it has anti-obesity and anti-cancer properties (De Meijer et al., 2003). Clinical studies have investigated the effects of Δ^9^-tetrahydrocannabinol (THC) on a variety of diseases, such as AIDS, advanced cancer, glaucoma, nausea, chemotherapy-induced vomiting, itching, allergies, psychiatric symptoms, and movement abnormalities (Barrales-Cureño et al., 2020). Some of these pharmacological potentials are summarized below in Figure 6.

### 5.1 Antimicrobial activities of *Cannabis sativa* extracts

Cannabinoids’ antimicrobial property has been known since the 1950s when the first reports appeared (Baswan et al., 2020). The bactericidal activity of *C. sativa* could not be linked to a single compound since these trials were performed before the phytochemistry of *Cannabis* was extensively known (Zheljazkov et al., 2020). It was accomplished in 1976 when it was discovered that both Δ^9^-THC and CBD are bacteriostatic and bactericidal against a panel of Gram-positive bacteria (De Vita et al., 2022). Antibacterial activities of *C. sativa* extracts, including essential oils and those obtained from petroleum ether, methanol, and hot water, have also garnered significant attention (Taghinasab and Jabaji, 2020). The oil from the seeds of the plant was extracted using petroleum ether and methanol and was found to exhibit antibacterial activity against Gram-positive bacteria. Interestingly, while the petroleum ether extract was found to be ineffective against *Pseudomonas aeruginosa*, it was observed to have some mild activity against Gram-negative bacteria (Ali et al., 2012). The principal components of *C. sativa* ethanol extracts showed moderate effectiveness exclusively against both clinical samples and non-clinical methicillin-resistant *staphylococcus aureus* (MRSA) infection isolates, as was earlier shown by Laidlaw (2016).

Figuerêdo et al. (2022) found that ethanol *Cannabis. sativa* seed extracts inhibited *S. aureus* biofilm development, suggesting that these extracts may have significant use as food and cosmetic preservatives. Also, Karas et al. (2020) discovered that cannabinoids are more successful than commercial toothpaste like Oral B and Colgate in reducing the bacterial colony count in dental plaque, suggesting that *C. sativa*-derived chemicals might be employed for oral care applications. Medical, aesthetic, veterinary, agricultural, and culinary uses of a Δ^9^-THC-free essential oil of *C. sativa* are all possible and are now being researched. Naringenin, a flavanone, was shown to contribute to the oil’s mild antibacterial efficacy and antibiofilm activity when tested against many strains of *S. aureus* (Zengin et al., 2018). *Helicobacter pylori*, a Gram-negative bacteria, was likewise shown to be susceptible to the antimicrobial effect, although no antifungal activity was detected (Fufa, 2019).

Several *S. aureus* and *Streptococcus* isolates were shown to be susceptible to CBD and Δ^9^-THC, with MIC in the range of 1–5 g/mL (Lohar and Rathore, 2013). This bactericidal action, however, was diminished in the presence of horse serum, most likely because of the cannabinoids binding to plasma proteins (Lohar and Rathore, 2013). Blaskovich et al. (2021) examined a variety of cannabichromene analogues for their antimicrobial and antifungal effects. Activity against *B. subtilis* and *S. aureus* seems to depend on the n-pentyl chain meta to the alcohol group (Blaskovich et al., 2021).

In addition to reducing biofilm formation, the use of certain *Cannabis*-infused medicines was found to modify other biofilm-associated virulence factors such as cell aggregation, hydrophobicity, membrane potential, and spreading ability (Feldman et al., 2018). These medicines can be used in conjunction with standard antibiotics like ampicillin and gentamicin to treat MRSA biofilm infections that have shown resistance to other treatments. CBD has also been shown to enhance the antibacterial action of the peptide medication bacitracin against *Staphylococcus species, L. monocytogenes*, and *E. faecalis* (Wanmakok et al., 2018). Several *Cannabis* analogues were tested by Wassmann and Klitgaard (2021) and proved to be effective against MRSA USA300 and *E. coli*. Several commonly used cannabinoids showed moderate to excellent activity; these findings were generally consistent with those of prior research (*vide supra*)*.* The MIC values increased by up to a factor of four (Ranieri et al., 2015).

CBD was also found by Sionov and Steinberg (2022) to be a synergistic agent when combined with different antibiotics. CBD was found to significantly inhibit the release of membrane vesicles by Gram-negative pathogens, which are involved in bacterial communication. When used in conjunction with erythromycin, vancomycin, rifampicin, kanamycin, or colistin, CBD’s antibacterial action was amplified against *E. coli* VCS257. These findings suggest that these cannabinoids may be used to increase efficiency and broaden the action of currently available antibiotics, which is an important development in the field of antibiotic resistance.

The *Cannabis* plant and its secondary metabolites have also been studied for their antifungal capabilities. Some articles claim that *Cannabis* extracts may be effectively utilized in the control of pathogenic fungi, albeit this impact has not been as widely explored as its antibacterial properties (Głodowska and Łyszcz, 2017). Popescu-Spineni et al. (2021) showed that ethanol and petroleum extract from *Cannabis* leaves effectively inhibited the growth and development of *Candida albicans*, *Candida krusei,* and *Aspergillus niger*. In both instances, the concentration of the leaf extract was 10 times greater compared to the antifungal antibiotic (Nystatin), although the zone of inhibition was substantially larger with the antifungal antibiotic. The antifungal properties of *Cannabis sativa* L. seed oil and whole-plant extracts of petroleum ether and methanol were investigated by Głodowska and Łyszcz (2017). However, while the whole-plant petroleum ether extract exhibited some action against *C. albicans*, the seed extract and whole-plant methanol extract were ineffective against the two fungi tested. For their ability to prevent the spread of the seed-borne phytopathogenic fungus *Alternaria* spp.; Akhtar et al. (2016) examined the antifungal properties of extracts from 11 weed varieties. Although all plants showed some antifungal activity, some were far more effective than others. The percentage of mycelial development that *Cannabis sativa* L. was able to halt was not the highest among the plants analyzed, but it was still rather high. The acetone-based extract proved to be the most effective antifungal agent among 5 distinct extract types. Some properties are summarized in Table 5 below.

**TABLE 5:** Antibacterial potentials of *Cannabis*.

S/N	*Cannabis* material	Solvent used	MIC	Type of assay	Pharmacological activity	Country	Reference
1.	Hemp essential oils	Methanol	0.25-32 µg/ml	MIC	Antibacterial activity	Italy	Iseppi et al. (2019)
2.	Hemp fiber	Acetone	5.64 x10^-7^g of antibiotics/100 mg of ester)	MIC	Antibacterial activity	Italy	Cassano et al. (2013)
3.	Oil of seeds, whole plant extract	Methanol	12.5-50 µg/ml	MIC	Antimicrobial activity	Sudan	Ali et al. (2012)
4.	Leaf extract	Distilled water	5.60-25.44 mm	Agar well diffusion assay	Antibacterial	India	Chouhan and Guleria (2020)
5.	lipophilic extracts of *Cannabis* products	propylene glycol	0.00125-1.25 μg/ml	MIC	Antibacterial activity	U.S.A	Gildea et al. (2022)
6.	Leaf extract	Ethanol, hot water	9.2-25.7 mm	Agar well diffusion assay	Antibacterial activity	Pakistan	Naveed et al. (2014)
7.	Aqueous leaf extract	Water	6-13 mm	Agar well diffusion assay	antibacterial activity	Romania	Csakvari et al. (2021)
8.	Cannabinoids	acetone	5 mg/ml	MIC	Antibacterial activity	Italy	Appendino et al. (2008)
9.	Cannabigerol	Aqueous	4 μg/ml	MIC	Antibiotic activity	Canada	Farha et al. (2020)
10.	Leaf extract	Ether and acetone extract	42 μg/ml	MIC	Bactericidal activity	India	Farha et al. (2020)

### 5.2 Anticancer activities

**FIGURE 7:**
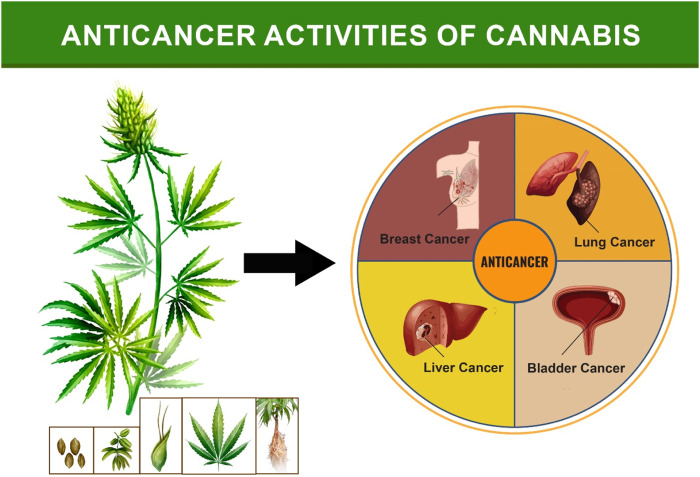
Anticancer activities of the *Cannabis*.

Cannabinoids (CBs) are active metabolites in *Cannabis sativa*, and they are responsible for the plant’s medical effectiveness (Kumar et al., 2021). CB derivatives have been shown to suppress the growth and survival of multiple forms of cancer cells. The underlying processes of the effects may be unique to each type of cell, and CBs can target tumors specifically to disrupt signaling and biological processes, leading to growth pause, cell death, and migratory blockage (Alexander et al., 2009). CBs may also have indirect effects on the tumor microenvironment, immune response, and vascularization suppression. Both direct and indirect anticancer effects of CBs have been studied (Hellmich and Szabo, 2015). In recent decades, there have been significant studies on the purity, efficacy, and therapeutic utility of *Cannabis* and cannabinoids (CBs) in preclinical and clinical cancer models. CBs have shown promise in treating and diagnosing cancer-related symptoms. THC, a type of CB, has been observed to accelerate the death of tumor cells compared to healthy cells. Long-term rat models have shown that Δ^9^-THC exhibits little toxicity and has no discernible impact on hematological parameters, general health, or mortality (Russo, 2016; Hartsel et al., 2019). Non-psychoactive CBD has been studied as a potential anticancer drug due to its action *in vitro* and *in vivo* against tumor cells produced from CBs found in *C. sativa*. Meanwhile, THC was administered to terminal cancer patients. However, the precise chemical pathways by which CBs exert their anticancer effects are not completely understood suggesting that more research involving the antitumor effects of CBs should be done. (Chung et al., 2021; Kumar et al., 2021). Secondly, regulation of the proinflammatory nuclear factor kappa B pathway has been linked to a tumor’s prosurvival impact, as well as chemoresistance in cancer cells, although the route is independent of Akt. Epidermal growth factor (EGF) signaling activation is essential for tumor cell growth, survival, and progression (Lau et al., 2018). In addition to its ability to destroy cancer cells, *Cannabis sativa* extract has anti-nausea and anti-vomiting properties that are beneficial for cancer patients. CBD has been found to reduce proinflammatory pathways by decreasing EGF signaling pathway activity (Pellati et al., 2018). By activating the TRPV channel and increasing endoplasmic reticulum stress, CBD can induce cancer cells to self-destruct. CBD also binds to and activates GPR55, which suppresses ERK pathway activation and halts cancer cell growth. As a result, multinational corporations are now offering medications containing cannabinoids in the form of plant extracts or volatile oils. Sativex, a standardized extract of *Cannabis* sativa L., has been licensed in Canada for the treatment of pain (Farag and Kayser, 2015). Various anticancer activities of cannabis found in liver, breast, bladder and lung are summarized below (Figure 7).

#### 5.2.1 Liver cancer

Liver cancer is a leading cause of mortality and suffering worldwide (Torre et al., 2015). CBs have been found to have anticancer effects by triggering apoptosis and suppressing telomerase activity. Low molecular weight hemp peptides have been shown to induce apoptosis, decrease cell viability, and reduce cell motility in Hep3B human liver cancer cells without modifying the baseline overexpression of cleaved caspase 3 and Bad, or downregulation of antiapoptotic Bcl-2 (Salamat et al., 2022). This strategy induced Akt and GSK-3 phosphorylation, followed by downregulation of β-catenin, demonstrating that β-catenin’s signaling modulation is the mechanism governing the anticancer activity. While further research is needed, these results suggest that hemp peptides may serve as a promising therapy for liver cancer (Kocatürk et al., 2021).

#### 5.2.2 Breast cancer

Breast cancer is the deadliest disease affecting females worldwide (Akram et al., 2017; Alsaraireh and Darawad, 2019). CBs have been found to significantly inhibit the proliferation of breast cancer cell lines, including human MDAMB231-luc-D3H2LN cells, which are sensitive to both 9-THC and CBD. CBs inhibited tumor invasion and metastasis in animal models and reduced EGF-induced proliferation and chemotaxis in triple-negative breast cancer cells (Arkell et al., 2019). CBD inhibited Id-1 receptor expression, cell proliferation, and invasion in breast cancer cells. In an athymic nude mouse model of breast cancer cells, CBD and CBG decreased tumor volume and promoted apoptosis. Δ^9^-THC was also found to reduce the growth of breast cancer cell lines (Kumar et al., 2021). At a concentration of 5 mM, CBD destroyed breast cancer cells in cell culture by cell-autonomous apoptosis and autophagy without harming normal cells, with relatively insignificant effects on TRPV1, CB1, and CB2 receptors (Perucca, 2017; Gaston and Szaflarski, 2018).

CBD’s *in vivo* antimetastatic activity was evaluated in syngeneic BALB/c mice by injecting 4 T1 breast cancer cells into the tail vein. CBD at 1 and 5 mg/kg suppressed both primary tumor growth and the number of metastatic foci by regulating cell migration through the activation of the ERK enzyme (Zhelyazkova et al., 2020). CBD at 5 mg/kg body weight also suppressed tumor development and lowered tumor volume in athymic nude mice with breast tumor xenografts, increasing the animals’ median survival time (Sakarin et al., 2022).

#### 5.2.3 Bladder cancer

Smoking cigarettes is a major contributor to the development of bladder cancer (Brennan et al., 2001). Numerous polls have found that a large percentage of cigarette smokers also regularly consume *Cannabis* (Hindocha et al., 2021). To determine the link between *Cannabis* and cigarette use and the development of bladder cancer in men in California, Thomas et al. (2015) conducted epidemiological research. They interviewed 84,170 males to find out about their habits like smoking and using *Cannabis*. The study indicated that while smoking alone was linked to a 15% increased risk of getting bladder cancer, *Cannabis* use alone was linked to a 45% lower risk. However, more rigorous studies are needed to thoroughly assess the plant’s medical potential in the treatment of bladder cancer.

#### 5.2.4 Lung cancer

Small-cell and non-small-cell lung cancers are the most common forms of lung cancer, with tobacco use, family history, and exposure to radon gas increasing the likelihood of developing the disease (Zou et al., 2021). CBs have been studied *in vivo* for their efficacy against lung cancer, and CBD therapy has been found to inhibit tumor growth, invasion, and metastasis in mice bearing xenografts of A549 cells (Solinas et al., 2015; Laezza et al., 2020). *In vitro*, investigations using lung cancer cell lines A549, H358, and H460 showed that CBD upregulated the antimetastatic protein ICAM-1, which is hypothesized to reduce tumor development through an immunosurveillance mechanism (Benedicto et al., 2017).

CBD was found to increase TIMP-1 and ICAM-1 expression in a dose-dependent manner in lung cancer cell lines and inhibited the spread and invasion of human lung cancer xenografts in mice, partially due to the increase in ICAM-1 and TIMP-1. It triggered apoptosis through PPAR-g and COX-2 in human metastatic lung cancer cells and caused tumor regression in A549 xenografted mice (Mrowka and Glodkowska-Mrowka, 2020). In human metastatic lung cancer cells and cancer cell lines A549 and H460, CBD and THC boosted ICAM-mediated lymphokine-activated killer cell adhesion and cancer cell lysis, increasing the lung cancer cells’ susceptibility to being lysed by LAK cells. Further research is needed to confirm *Cannabis*’ protective effect against lung cancer (Seltzer et al., 2020).

### 5.3 Epilepsy

As a neurological condition, epilepsy is characterized by aberrant brain activity and frequent seizures (Rana and Musto, 2018). During the first decade of life, this affects about 1 in 150 children (Ramantani et al., 2013). Epileptic encephalopathies are characterized by refractory seizures, severe electroencephalographic abnormalities, and developmental impairment (Khan and Al Baradie, 2012). Clinical evidence for the use of CBs in the management of epilepsy has been backed up by preliminary studies (Antonarakis et al., 2020; Oberbarnscheidt and Miller, 2020). Ellen et al. (2018) reported that using CBs in a mouse model of Dravet syndrome led to a decrease in autistic-like social deficiencies, suggesting that these drugs’ benefits extend beyond seizure control.

Clinical investigations have shown that CBD and cannabidivarin (CBD’s propyl version) have anticonvulsant qualities, although the particular processes by which they do so remain unknown (Devinsky et al., 2014). A large, prospective, single-center, open-label study of CBD for the treatment of medication-resistant epilepsy in children and adults showed striking improvements in disease phenotype in response to CBD therapy for 72 children and 60 adults (Sher and Maldonado, 2015). In addition, EPIDIOLEX^®^, a CBD medicine derived from *Cannabis*, was recently licensed by the FDA for the first time to treat Lennox-Gastaut syndrome and Dravet syndrome, two extremely uncommon but extremely serious forms of epilepsy (Lattanzi et al., 2021). These results suggest that CBs including CBD may be useful for the treatment of epilepsy and other neurological disorders (Kaplan et al., 2017). Furthermore, CBD proved beneficial in reducing seizure frequency in a comprehensive trial and meta-analysis of its efficacy for treatment-resistant epilepsy (Pamplona et al., 2018).

### 5.4 Parkinson’s disease

There are motor and non-motor symptoms associated with this kind of neurodegenerative disease. Non-motor symptoms of Parkinson’s disease, such as constipation, sleep issues, anxiety, and sleep instability, were studied by Sauerbier et al. (2016), who reviewed the data of many trials to determine whether CBD may be helpful. Dopamine-containing neurons in the basal ganglia were shown to deteriorate in Parkinson’s disease, which may be linked to mitochondrial malfunction, oxidative stress, and impaired protein breakdown in the affected cells (Poewe et al., 2017). Several studies have found a correlation between the endocannabinoid system (ECS) and Parkinson’s disease (Han et al., 2020). The ECS consists of cannabinoids (CB) receptors, i.e., CB1 and CB2, their ligands, and the enzymes responsible for their production and metabolism (Iannotti et al., 2016). The basal ganglia of the brain are where endocannabinoids are most concentrated. By activating or inhibiting CB1 or CB2, CBD contributes to the lowering of dopamine levels. CBD’s sedative action has also been studied, with mixed results (depending on dosage and mode of administration) including enhanced sleep delay and alertness (Sarris et al., 2020). Rats given either high or moderate dosages of CBD in an experiment by Silvestro et al. (2019) slept longer and for longer periods thereafter.

One study found the effectiveness of *Cannabis*-based medicinal extracts v/s placebo for the treatment of individuals with spinal cord injury (SCI) (Grotenhermen, 2004). Peppermint oil, 0.05% (v/v), ethanol, and propylene glycol (50:50) were the excipients in a THC (27 mg/mL): CBD (25 mg/mL) extract of *Cannabis sativa* L. After receiving treatment, patients reported significantly higher ratings of central neuropathic pain on the 11-point numerical rating scale, with a negative number indicating an increase in pain from pre-treatment levels. Thomas et al. (2021) also did a scoping assessment of the literature on *Cannabis*’s effect on SCI pain severity. Variations in methodology, such as the lack of standardized dosage regimens, modes of use, and trial length, led to contradictory findings across the study’s articles reporting on five treatment studies. Consistent and sufficient data is, therefore, lacking to form accurate conclusions on the efficacy of *Cannabis* in lowering the pain intensity associated with SCI, indicating that more study is needed in this area.

### 5.4 Gastrointestinal disorders

*Cannabis* is used by inflammatory Bowel Disease (IBD) patients to alleviate symptoms and improve their quality of life. Endocannabinoids (ECS) help in maintaining intestinal homeostasis, which requires a combination of centrally and peripherally mediated actions (Russia et al., 2015). The ECS consists of endocannabinoids, enzymes that make and break down endocannabinoids, and CB receptors that mediate endocannabinoid effects (Kilaru and Chapman, 2020). The enzymes responsible for the breakdown of endocannabinoids are fat acid amide hydrolase (FAAH) and monoacylglycerol lipase (MGL). However, more research is needed to understand the role of the ECS in IBD and the effects of *Cannabis* on these conditions (Gil-Ordóñez et al., 2018).

The endocannabinoid system (ECS) is present throughout the gut and controls several digestive processes such as GI motility, inflammation, and immune response. *Cannabis* can potentially impact these processes by activating the receptors in the ECS, leading to an increase in food intake and metabolic processes like lipolysis and glucose metabolism (Sergi et al., 2021).

*Cannabis* is used for various gastrointestinal (GI) ailments, including enteric infections, inflammation, motility difficulties, emesis, and stomach discomfort. Endocannabinoids can inhibit proinflammatory mediators such as IL-1β, TNF-α, and nitric oxide, reducing the cellular pathways leading to the coordinated inflammatory reactions in IBD (Saqib et al., 2017; Bennett et al., 2018). *Cannabis* formulations have been shown to significantly reduce the severity of colitis in experimental animal models of IBD. CBs can regulate GI motility, which has paved the way for the development of a new class of antibiotics that can treat a wide range of GI conditions, including colitis, Crohn’s disease, gastric ulcers, paralytic ileus, IBS, colon cancer, and others (Tyakht et al., 2018).

The ECS can be useful in managing irritable bowel syndrome-diarrhea (IBS-D) and irritable bowel syndrome-constipation (IBS-C) by affecting motility and secretion through CB1 agonists. CB2 receptors can be used to treat IBS-D because they are overexpressed during stomach inflammation (Uranga et al., 2018). The ECS plays an inhibitory role in the GI tract by suppressing motility and secretion and controlling pain perception. CB receptor activation can protect against colitis, and inhibiting breakdown enzymes (FAAH or MAGL) can reduce inflammation. Inhibiting the enzyme that produces 2-AG (DAGL) can regularize feces in constipation-prone animals and reduce 2-AG levels (Karwad et al., 2017).

Studies have shown that patients with Crohn’s disease have increased CB receptors and/or endocannabinoids in their intestines. CB1 and/or CB2 agonist treatment has been found to reduce colitis in animal models of inflammatory bowel disease (Hryhorowicz et al., 2021). Clinical research on the use of Δ^9^-THC to treat Crohn’s disease has shown promising results, but further studies are needed to determine appropriate doses, modalities of usage, patient populations that would benefit, and long-term exposure risks through randomized, controlled, and prospective clinical studies (Ziemssen and Thomas, 2017).

## 6 *Cannabis*’ potential in the food industry

According to Kanabus et al. (2021), *Cannabis* cultivars can be used in food production in Europe if the amount of ∆^9^-THC and ∆^9^-THCA in unrecognized blooming or fruiting plant tips is less than 0.2% dry matter. This requirement is in place to prevent the production of edible *Cannabis* products, but the consumption of hemp seeds is still allowed. In November 2015, the European Union passed Regulation 2015/2283, which designates certain hemp extracts and parts as “novel foods" (Kanabus et al., 2021).

There’s a wide variety of baked goods, pizza, oil, beer, milk, chocolate, ice cream, and snacks made with *Cannabis* seeds (Kanabus et al., 2021; Sorrentino, 2021). *Cannabis*-related food exports from Italy are worth 50 billion euros and make a big difference in the country’s economy (Sorrentino, 2021). The hemp-based agri-food chain may be the biggest step forward for this industry. Demand for hemp-based foods has increased fivefold from 2017 levels (Moliterni et al., 2022). Hemp seed flour is a healthy alternative to wheat flour. Although it tastes rough and rustic, hemp flour has 21% fewer calories than regular oat flour (Albala, 2003). Celiacs can eat it without worry because it does not contain gluten (Sorrentino, 2021). According to Sorrentino (2021), hemp has only 25% protein and 65% edestin. Recent studies by Bao et al. (2014) have shown that, like animal proteins, edestin provides all eight essential amino acids. These findings reveal that *Cannabis* has the potential to make the world’s food supply much safer by lowering the need for animal protein.

## 7 *Cannabis*’ potential in the Cosmetics industry

*Cannabis* seeds are frequently used in traditional cosmetic treatments, especially for hair care, due to their high oil content. Michailidis et al. (2021) studied the effect of *Cannabis* seed oil on the strength of hair and nails. *Cannabis* stems are also highly valued, particularly in Pakistan (Michailidis et al., 2021).

Moreover, *Cannabis* seed oil is often used as a hair food due to its rich nutritional profile. The oil is particularly high in polyunsaturated fatty acids, such as omega-3 and omega-6 fatty acids, which are essential for maintaining healthy hair (Crini et al., 2020). These fatty acids help to nourish and moisturize the hair, making it softer, smoother, and more manageable. They also help to prevent hair breakage, split ends, and dryness, which can lead to hair loss over time (Callaway, 2004).

In addition to its fatty acid content, *Cannabis* seed oil is also high in carotenoids, which have been shown to promote hair growth and improve hair health (Baral et al., 2020). *Cannabis* seed oil can be used in a variety of ways as a hair treatment. It can be applied directly to the scalp and hair as a hair mask, left on for several minutes or overnight, and then rinsed off with shampoo and conditioner. The oil can also be added to shampoos and conditioners to enhance their moisturizing and nourishing properties (Baral et al., 2020). Carotenoids, such as β-carotene, can help to strengthen the hair shaft and protect it from damage caused by UV radiation and other environmental stressors. They can also help to improve the elasticity and overall appearance of the hair (Nahhas et al., 2019).

The endocannabinoid system (ECS) in the skin plays an important role in regulating cell differentiation, development, survival, inflammation and immune responses, pain perception, and hair growth (Mnekin and Ripoll, 2021). Disruption of the ECS can lead to various dermatological problems (Del Río et al., 2018). The ECS is controlled by CB1 and CB2 receptors, which are found in different cells in the skin. CB1 is expressed in hair follicle cells, immune cells, and keratinocytes and regulates pain, neuronal activity, and inflammation. CB2 is found in sensory neurons, immune cells, sebaceous glands, and keratinocytes and also regulates inflammation (Baswan et al., 2020). Activating CB1 has been shown to prevent keratinocytes from producing pro-inflammatory cytokines and maintaining the integrity of the epidermal barrier (Gaffal et al., 2013).

*Cannabis* seed oil is a rich source of carotenoids, such as β-carotene, lutein, and zeaxanthin, which are easily absorbed by the skin (Irakli et al., 2019). These carotenoids have antioxidant properties that can combat free radicals and protect against UV light (Wisniewska et al., 2006). β-carotene can also prevent the activation of pro-inflammatory cytokines by UV-B radiation, thus exhibiting anti-inflammatory effects. Carotenoids can also improve skin hydration, promote wound healing, and stimulate the production of collagen and elastin by activating fibroblasts (Baswan et al., 2021). *The Cannabis* seed oil has a high concentration of chlorophyll, which can range from 100 μg/g to 230 μg/g, depending on the extraction process (Stephens et al., 2015). Chlorophyll has been shown to promote tissue growth and have antibacterial properties, making it potentially useful in wound healing and treating skin problems like acne, eczema, and ulcers. The green pigment in hemp seed oil comes from chlorophyll (ElSohly, 2007).

**FIGURE 8:**
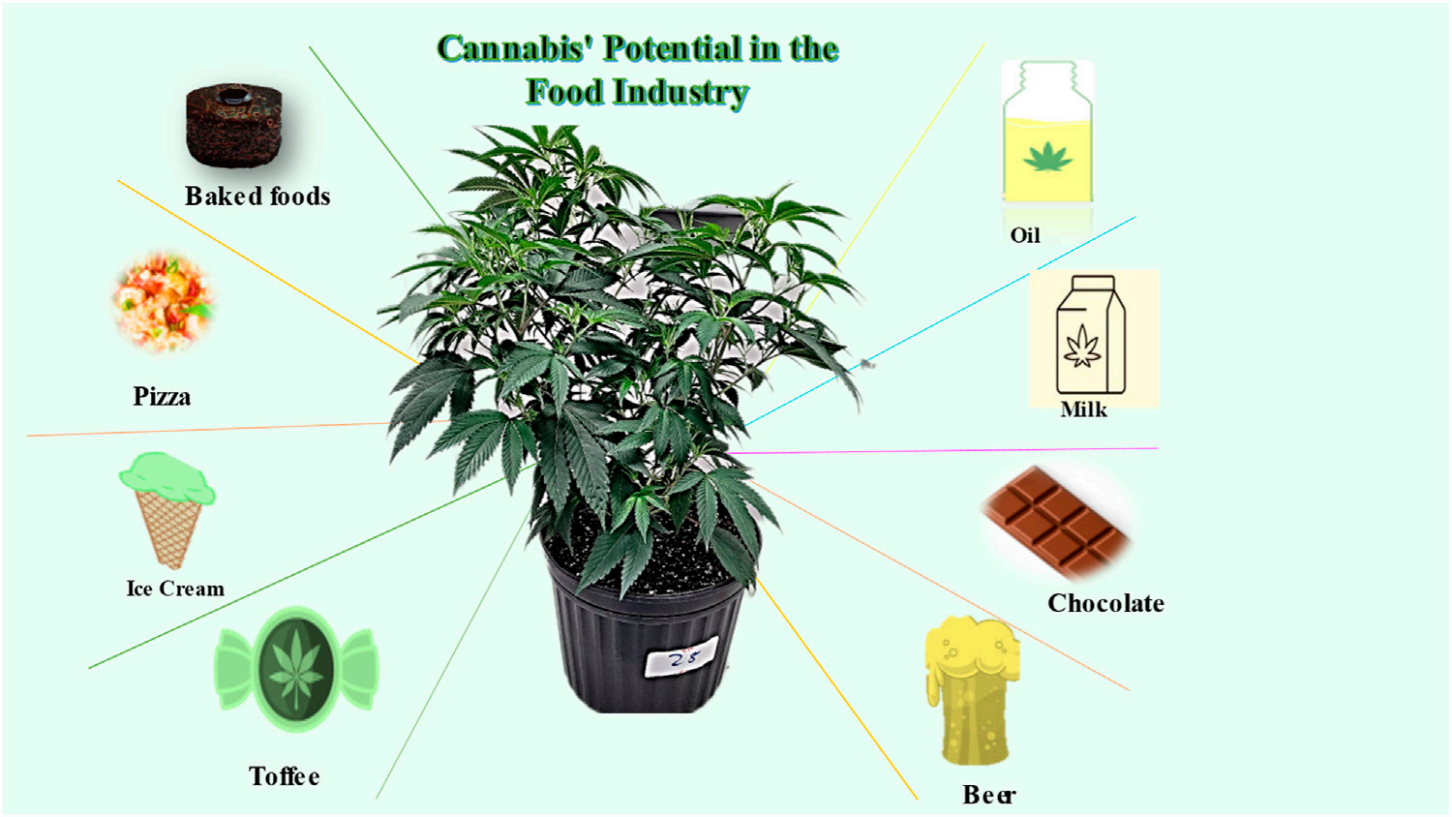
*Cannabis* potential in the food industry.

The *Cannabis* seed oil contains flavonoids, terpenes, carotenoids, chlorophylls, and phytosterols that contribute to its anti-inflammatory and anti-aging properties. The oil is quickly absorbed and does not clog pores, making it useful in formulations designed to soothe the skin, such as sunscreen creams and lotions. The natural presence of chlorophyll makes it potentially effective for wound healing and treating skin problems. Topical creams and ointments containing *Cannabis* seed oil have potential applications in anti-aging skincare (Baral et al., 2020). The various uses are summarized below in Figure 8 below.

## 8 Significance of *Cannabis* cultivation to agriculture and environment

*Cannabis* sativa L. was first cultivated for textile fiber in Western Asia and Egypt. It later spread to Europe and was eventually brought to North America in 1,606, beginning with Port Royal in Canada (Small and Marcus, 2002). *Cannabis* farming has a low negative environmental impact because it can proliferate, kill weeds, and does not require pesticides. It does not have parasites that are only beneficial for one plant, which helps with pollination and improves soil fertility (Zheng et al., 2021). Although *Cannabis* sativa was traditionally used as a source of stem fiber and was rarely considered a narcotic, it has been one of the world’s oldest sources of textile fibers for more than 6,000 years. Its use as an oil crop was limited for most of its existence (Small, 2015).

Hemp was introduced for fiber production in Western Asia and Egypt between 1,000 and 2000 BC and later spread to Europe. After 500 AD, the cultivation of hemp became widespread throughout Europe (Clarke and Watson, 2002). Clarke and Merlin (2013) also provide an excellent overview of the historical and cultural use of *Cannabis*. *Cannabis* is considered an environmentally friendly crop in recent times, with added interest in its cultivation due to its potential to help combat climate change and desertification. As a result, the EU has proposed *Cannabis* cultivation as a potential new star in European agriculture, aligning with EU 2030 goals of reducing greenhouse gas emissions by 40% from 1990 levels. (Sorrentino, 2021; Zheng et al., 2021).

*Cannabis* can reduce the amount of Carbon dioxide (CO_2_) in the atmosphere, and it is particularly effective due to its high rate of growth. This makes it a valuable agricultural species for reducing greenhouse gases. However, the current atmospheric CO_2_ level is still much higher than pre-industrial levels, so further research is needed to find more effective ways to reduce carbon emissions (Showalter et al., 1996; Thomas and Elsohly, 2015). The use of slow-release nitrogen fertilizers in which urea is combined with an aldehyde like nitroform, methylene urea, or urea formaldehyde is recommended for *Cannabis* farming due to their positive impact on plant growth and seed quality (Butsic et al., 2018). In contrast, the use of synthetic fertilizers like ammonium nitrate can increase greenhouse gas emissions like nitrous oxide (N_2_O), which contributes to global warming and is a significant source of emissions in some countries (Sorrentino, 2021). *Cannabis* has a different eco-physiological trait than cotton and kenaf, where it is not as efficient in using nitric nitrogen. However, it excels at photosynthetic metabolism at low nitrogen levels (Dilley and Morrison, 2014). Slow-release nitrogen fertilizers, such as urea-formaldehyde, can reduce the amount of N_2_O released during the growth cycle, which is a significant contributor to greenhouse gases. Additionally, *Cannabis* stores CO_2_ in its biomass, making it a potentially climate-friendly crop that can help prevent climate change (Stone, 2011).

Growing *Cannabis* has the potential to set up new supply chains due to the versatility of the different plant parts. This could be beneficial for farmers, the environment, and human health, making it an important plant for the new green economy (Sorrentino, 2021). Various industries can use different parts of the plant: seeds for the agri-food industry, canapulo for the green building sector, fiber for the textile industry, and inflorescences and roots for the pharmaceutical and para-pharmaceutical industry through the extraction of bioactive molecules (Akhtar et al., 2016).

*Cannabis* requires nutrients and water to grow, with varying daily water use depending on location, soil, weather, and growing methods (Carah et al., 2015; Zheng et al., 2021). Outdoor *Cannabis* cultivation in California uses an average of 5.5 gallons of water per day per plant, according to a survey (Wilson et al., 2019). Agricultural usage, population growth, and climate change are expected to worsen water shortages, which will affect the *Cannabis* industry and harm the environment (Schlenker et al., 2007; Zheng et al., 2021). The amount of water needed for *Cannabis* plants to survive and thrive is a concern for the industry (Moyle, 2002). *Cannabis* cultivation, particularly illegal cultivation, can lead to water contamination. The plant requires increased levels of nitrogen, phosphorus, and potassium for optimal growth, but little research has been done on how this affects water quality globally (Saloner et al., 2019). The use of pesticides, such as herbicides, insecticides, fungicides, nematocides, and rodenticides, can also contribute to water contamination when not properly checked, posing a threat to the environment (Gabriel et al., 2013). *Cannabis* cultivation can lead to the contamination of soil, surface water, and groundwater due to the leakage of nitrogen and pesticides from runoff or rain (Thompson et al., 2014). This can harm both humans and crops that consume these chemicals. The contamination of water caused by *Cannabis* cultivation can also impact the environment where other important irrigated crops are grown (Thompson et al., 2014). However, it is difficult to link *Cannabis* farming practices to water pollution without proper measurement of water quality and chemical levels. Thus, legislation is needed to protect the environment from pollutions arising from commercial *Cannabis* cultivation. In this regard, Canada has some of the strictest environmental regulations for growing *Cannabis* indoors to mitigate the impact on the environment.

Zheng et al. (2021), report that *Cannabis* production is directly linked to soil erosion, especially on steep slopes that are more prone to erosion. The cutting down of trees and clearing of forests for *Cannabis* cultivation exacerbates soil erosion. However, durable greenhouses can help prevent soil erosion by avoiding the need for massive clearings that expose soil to erosion (Bauer et al., 2015).

*Cannabis* can help address biodiversity loss by attracting pollinating insects due to its terpenoid essence, and its pollen can be blown up to 3 km, increasing the diversity of agroecosystems (Sorrentino, 2021). The plant blooms at different times and provides ample pollen, making it an important source of food for bees (Balcke et al., 2014). By creating a microclimate that is beneficial for pollinators, *Cannabis* contributes to the conservation of biodiversity, which is essential to the health of the planet (Flicker et al., 2020). Another potential application of *Cannabis* is a bioremediation crop that can absorb and store heavy metals from the soil, making it effective in cleaning contaminated soil (McPartland and McKernan, 2017). Tainted soil fertilizer is a common source of arsenic, cadmium, lead, and mercury. Singani and Ahmadi (2012) found that *Cannabis sativa* may absorb lead and cadmium from manure-contaminated soils.

## 9 Risk and safety of *Cannabis*

The widespread acceptance of medical and recreational *Cannabis* usage in recent years has contributed to a surge in the drug’s popularity in several countries (Hajizadeh, 2016). There is an ongoing debate among experts regarding the safety of the plant. While in some parts of the world, it is viewed as a helpful medicine, in other parts it is seen as a dangerous substance if taken in large amounts, particularly when it comes to the consumption of *Cannabis*-containing foods or beverages (Lindblom, 2019). Research suggests that certain genetic variants may increase the risk of mental health problems in individuals who use *Cannabis* due to its impact on brain development, including neuroanatomical alterations, respiratory problems, metabolic and neurotransmitter functioning, and neuronal activation (Gage et al., 2016; Connor et al., 2021).

Second, *Cannabis* has been linked with negative effects on conditions like cardiopulmonary arrest, coronary artery disease, transient ischemic attack, and cannabis arteritis. Additionally, exposure to high amounts of THC for recreational use has been found to negatively affect various physiological systems, including ophthalmological, gastrointestinal, respiratory, immunological, and hormonal systems (Le Boisselier et al., 2017). However, serious poisoning is rare among adults and negative side effects are reported by only a small percentage of users. A study found that only 3.24% of participants reported negative side effects from using *Cannabis*. The flower was used in 42.86% of cases, and the leaf was used in 40.8% of cases. The study identified 45 different side effects, but only one mention of death (Boakye et al., 2021).

Furthermore, ancient medicine recognizes both the benefits of *Cannabis* and the risks of overuse (Iber et al., 2022). Research has found that *Cannabis* has both stimulating and sedative effects, including increased appetite and aphrodisiac effects as well as the ability to cool the body (Seltenrich, 2019). However, according to Volkow et al. (2014), long-term use can have unintended side effects such as stomach pain, thinning skin, depression, inability to work, and dropsy (a buildup of water in the body) (Volkow et al., 2014). Using the right amount of *Cannabis* is important for both medical and recreational purposes. In 2021, the United Nations Office on Drugs and Crime (UNDOC) removed *Cannabis* from Schedule IV, but it remains on the Schedule I list due to insufficient data on its effects (Riboulet-Zemouli and Krawitz, 2022). For instance, reports have linked *Cannabis* use to the growth of tumors, including in children whose mothers’ used marijuana during pregnancy. Depending on the dose and length of use, *Cannabis* can also cause cancer, birth defects, and genetic changes. In addition to harming mental health, *Cannabis* use can negatively impact respiratory, cardiovascular, and bone functions (Sharma et al., 2012).

One of the main risks associated with *Cannabis* use is the potential for overconsumption, particularly when consuming edibles or other foods infused with *Cannabis* (Lin et al., 2022). This is because the effects of ingested *Cannabis* can take longer to manifest and last longer as compared to when *Cannabis* is smoked or vaporized, leading users to inadvertently consume more than intended (Grant and Bélanger, 2017). Overconsumption of *Cannabis* can cause severe side effects, including vomiting, nausea, anxiety, paranoia, and, in the extreme cases can lead to hospitalization (Ford et al., 2017). A study found that the rate of emergency department visits related to *Cannabis*-containing edibles increased significantly after legalization in Colorado (Heard et al., 2017).

Moreover, one of the major safety concerns associated with *Cannabis* use is the potential for contamination with biological, physical, or chemical contaminants (Rather et al., 2017). Microbial contamination of *Cannabis*-containing foods for instance can lead to foodborne illness, especially in persons with weak immune systems or other underlying health conditions. In one study, researchers analyzed a variety of *Cannabis*-infused food products and found that many were contaminated with high levels of bacteria including *E. coli* and *Salmonella*. In other studies, a significant percentage of *Cannabis* products tested were found to be contaminated with pesticides, mycotoxins, and heavy metals above the legal limit (Seltenrich, 2019; Peng and Shahidi, 2021; López-Ruiz et al., 2022). In addition to these risks, there is also the potential for adverse drug interactions between *Cannabis* and other medications, particularly those that are metabolized by the liver (Brown and Winterstein, 2019). *Cannabis* can interact with certain medications, such as blood thinners and antidepressants, leading to unintended side effects or reduced effectiveness of these drugs (Polson et al., 2021). Furthermore, there are concerns about the potential for *Cannabis* to interact with other medications or supplements (Wheeler et al., 2020). For example, a study found that consuming grapefruit juice with *Cannabis*-containing products can increase the levels of THC in the blood, potentially leading to an increased risk of adverse effects (Tireki, 2021).

Another issue is the lack of standardized dosing guidelines for *Cannabis*-containing foods (Nyland and Moyer, 2022). Because the potency of these products can vary widely, it can be difficult for consumers to know how much of a particular product they should consume to achieve the desired effects without risking overconsumption (Potter, 2014). This has led to instances of accidental overconsumption and adverse effects, particularly in the case of edibles, which can be deceivingly potent (Larkin Jr and Madras, 2019). Another concern is the potential for *Cannabis*-containing foods to be appealing to children and young people. As these products become more widely available, there is a risk that they could be mistaken for regular food items and ingested by children, potentially leading to serious adverse effects (Karbakhsh et al., 2018).

To address this issue, some jurisdictions have implemented packaging and labeling regulations for *Cannabis*-containing products to make them less appealing to children. In the United States, for example, the FDA requires that all *Cannabis*-containing food products be labeled with the statement “Keep out of reach of children” and include a warning that the product contains *Cannabis* (Nyland and Moyer, 2022). Some states such as Colorado, California, and Washington have gone further, requiring that products be packaged in child-resistant containers or that the packaging be opaque or non-descript to reduce their appeal to children. Similarly, in Canada, the *Cannabis* Act requires that all *Cannabis*-containing products be packaged in child-resistant containers and display a standardized warning label that includes the THC content and other relevant information (Leos-Toro, 2019). In addition, the act prohibits the use of branding and labeling that may appeal to children, such as cartoon characters or bright colors (Leos-Toro, 2019). In Australia, *Cannabis*-containing products must be packaged in opaque, child-resistant packaging and display warnings about the potential health risks associated with consumption (Rasera et al., 2021). In the Netherlands, all *Cannabis*-containing products must be labeled with a warning that they are not intended for consumption by children or minors (Rasera et al., 2021).

## 10 Conclusion and future perspectives

*Cannabis* is a versatile plant with many therapeutic uses. The current review has shown that it contains compounds with numerous therapeutic benefits, such as antioxidants, cytotoxic agents, and antibacterial, antifungal, anticancer, antidiarrheal, neuroprotective, and hepatoprotective properties. *Cannabis sativa* can be used in a variety of industries including biomedicine, agriculture, food, and cosmetics.

These bioactivities are attributed to its phytoconstituents, including cannabinoids, terpenes, flavonoids, alkaloids, and steroids, which highlight its potential as a source of medicinal agents. In addition to its medicinal uses, *Cannabis* has diverse applications in agriculture as fibre, food (as a source of protein, fiber, and functional foods), and cosmetics (as an active ingredient or oil). Although the available literature demonstrates the endless potential of *Cannabis* in these areas, more extensive research is needed to uncover the many unknown chemical substances with medical value. While empirical investigations of the plant have already been established, further studies should aim to identify and test these substances for their therapeutic benefits in treating various ailments.

In addition, *Cannabis sativa* L. is often overexploited as a recreational drug despite its potential uses in various fields due to its easy accessibility. Studies have confirmed its toxicity to brain development and the nervous system, but its extensive traditional use presents challenges in controlling its impact. Engaging users to understand the plant’s potential and training growers in cultivation, extraction, and production can help reduce overexploitation. Policies are also needed to protect and utilize the benefits of *Cannabis* plants. Community engagement, planning, monitoring, evaluation, and implementation can all help in this effort.
